# LncRNA *Dlx4os* drives malignant transformation and phenotype switching in melanoma

**DOI:** 10.1080/15592294.2026.2641924

**Published:** 2026-03-19

**Authors:** Ana Luisa Pedroso Ayub, Beatriz Cristina Biz Tonin, Hátylas Azevedo, Pedro Henrique Fogaça Jordão, Tanvi Saxena, Leinal Sejour, Jeremie Nsengimana, Ioannis Vlachos, Eduardo Moraes Reis, Frank John Slack, Miriam Galvonas Jasiulionis

**Affiliations:** a Department of Pharmacology, Escola Paulista de Medicina, Universidade Federal de São Paulo, São Paulo, Brazil; b Departament of Urology, Escola Paulista de Medicina, Universidade Federal de São Paulo (EPM/UNIFESP), São Paulo, Brazil; c Department of Biochemistry, Institute of Chemistry, Universidade de São Paulo (USP), São Paulo, Brazil; d Department of Pathology, Beth Israel Deaconess Medical Center, Harvard Medical School, Boston, MA, USA; e Biostatistics Research Group, Population Health Sciences Institute, Faculty of Medical Sciences, Newcastle University, Newcastle, UK

**Keywords:** Melanoma, lncRNAs, plasticity, phenotype switching, malignant transformation

## Abstract

Cutaneous melanoma, a highly aggressive and therapy-resistant skin cancer, is characterized by its remarkable cellular plasticity, enabling tumour cells to switch between different phenotypic states. This plasticity contributes to tumour heterogeneity and is regulated by key transcription factors. Long non-coding RNAs (lncRNAs) are emerging as crucial regulators in melanoma progression, yet much remains to be explored regarding their role in phenotype switching. In this study, we analysed long non-coding RNAs (lncRNAs) across different murine melanoma cell lines, identifying a set of lncRNAs potentially involved in regulating melanoma phenotypic state through cis-regulation of neighbouring protein-coding genes. We demonstrated that the lncRNA *Dlx4os* regulates genes associated with melanoma plasticity, favouring a mesenchymal-like, undifferentiated state. *Dlx4os* knockdown redirected melanoma cells to a more differentiated and less malignant phenotype, confirmed by differential expression of phenotypic state markers (*Sox10, Mitf, Tgfβ, Sox6, Mlana*), reduced their invasive and migratory potential, and delayed tumour progression *in vivo*. Furthermore, we identified a human orthologue of *Dlx4os*. Our findings highlight the potential of *Dlx4os* as both a biomarker and therapeutic target, capable of modulating melanoma’s phenotypic plasticity to influence treatment response and metastasis.

## GRAPHICAL ABSTRACT

**Figure:**
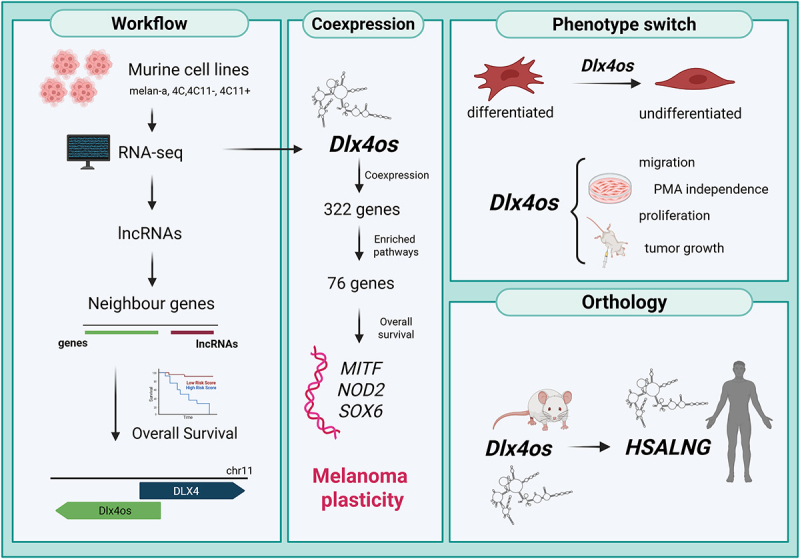

## Introduction

Human cutaneous melanoma, which accounts for approximately 90% of all melanoma cases, originates from the malignant transformation of melanocytes in the basal layer of the epidermis [1]. This type of skin cancer is characterized by its high metastatic potential and resistance to therapy [2]. One of the main characteristics of melanoma is its high cellular plasticity, which arises from its origin in neural crest-derived melanocytes. This plasticity allows melanoma cells to transit between different transcriptional states, a process referred to as phenotype switching [3,4], contributing to tumour heterogeneity in melanoma [5].

Recently, several transcriptional profiling studies have further resolved this heterogeneity, categorizing melanoma cells into distinct states, each with specific molecular subtypes (mesenchymal-like/undifferentiated; neural crest-like; intermediate; melanocytic differentiated and hyperdifferentiated subtype) [5–7]. All melanoma states are associated with specific regulator genes that act as ‘master regulators’ of this process. Key players in melanoma plasticity include *MITF*, the *SOX* family, *MLANA*, *TGFβ*, *SNAIL* and other genes, which define distinct melanoma states. These states arise because melanoma cells can adopt various characteristics, such as enhanced proliferation or invasion, resulting in subcellular phenotypes that are more prone to metastasis or that respond differently to treatments [8].

The ability of melanoma cells to adapt to various environments in a potentially reversible process through phenotype switching is a critical factor underlying their capacity for metastasis and therapy resistance [3], and it can be strongly regulated by stable yet reversible epigenetic reprogramming [8] in line with melanoma cells’ survival requirements.

Long non-coding RNAs (lncRNAs), a type of non-coding RNA, are complex molecules that can interact with epigenetic factors and play significant roles in complex biological processes [9], acting as central players in regulatory circuits [8,10]. Various lncRNAs have already been associated with phenotype switching, making cells more resistant or sensitive to therapy and altering melanoma aggressiveness through phenotype modulation [8]. However, there is still much to be explored, given the relatively low number of studied lncRNAs compared to the total number of non-coding RNAs [8]. Additionally, one of the challenges in studying lncRNAs is their diverse mechanisms of action [11,12], opening multiple possibilities of research in the field.

In this work, we conducted a systematic analysis of lncRNAs across different melanoma states to identify regulatory nodes involved in melanoma phenotypic switching. Initially, our approach focused on investigating lncRNAs through the cis-regulation of protein-coding genes with prognostic value in melanoma, generating a list of lncRNAs with potential roles in melanoma phenotypic switching. Furthermore, we selected the lncRNA *Dlx4os* and investigated its relationship with its neighbouring gene, *Dlx4*, before expanding our exploration to examine its capacity for gene trans-regulation. Here, we discovered that *Dlx4os* can modulate melanoma phenotype switching through trans-regulation, altering the melanoma cell state from nonmalignant to a mesenchymal-like/undifferentiated state, thereby promoting malignant transformation. Additionally, we conducted a pioneering analysis to identify a potential human homolog of *Dlx4os*, proposing a candidate that has never been described before. This discovery opens up novel avenues for exploring *Dlx4os* both as a drug target and a tumour biomarker. The detection of this lncRNA as a biomarker could serve as an approach for the early detection of melanoma and for characterizing the tumour based on its stage.

## Material and methods

### Cell lines representing different stages of melanoma progression

We used a multi-stage cellular model of melanoma progression developed by our group [13–29], in which the non-tumorigenic lineage of spontaneously immortalized murine melanocytes [30], named melan-a, was subjected to sequential cycles of substrate adhesion blockade. During the cycles, most cells died by adhesion blockade-induced apoptosis (*anoikis*); however, a few melan-a cells survived, and were collected and expanded under normal adhesion conditions, and then subjected to further cycles of adhesion blockade (two, three, and four de-adhesion cycles). After the last de-adhesion cycle, the spheroids formed by the 4C (four cycles) cells were subjected to limiting dilution, and different clones were randomly selected and expanded to generate distinct cell lines, including the 4C11-. The 4C11+ cell line was established from the 4C11- cell line after the spontaneous loss of p53 protein expression. Therefore, this linear model consists of different cell lines representing the main steps of melanoma progression: non-tumorigenic melanocytes (melan-a), premalignant melanocytes (4C), non-metastatic (4C11−), and metastatic (4C11+) melanoma cells. The *in vitro* characterization of the model has found that the 4C cell line resembles premalignant melanocytes (non-transformed, low proliferative, mesenchymal-like and undifferentiated cells), whereas 4C11− are non-metastatic, low proliferative, mesenchymal-like and undifferentiated melanoma cells, and 4C11+, metastatic, highly proliferative and differentiated melanoma cells [27].

### Cell culture

The cell lines melan-a, 4C, 4C11-, and 4C11+ were cultured in RPMI 1640 medium (Thermo Fisher Scientific, Waltham, MA, USA), pH 6.9, supplemented with 10 U/ml of Penicillin and 10 U/ml of Streptomycin (Thermo Fisher Scientific, Waltham, MA, USA), and 5% fetal bovine serum (Thermo Fisher Scientific, Waltham, MA, USA). For the melan-a cell line, phorbol myristate acetate (PMA) (Thermo Fisher Scientific, Waltham, MA, USA) was added to the culture medium (final concentration of 200 nM). The human cell lines WM35 (radial growth phase, RGP), WM983C (metastatic), were cultured in TU medium (MCDB and Leibovitz’s L-15 supplemented with insulin at 4.9 mg/mL and CaCl_2_ at 1.6 M, pH 6.9), containing 2% fetal bovine serum and 10 U/mL each of penicillin and streptomycin (Thermo Fisher Scientific, Waltham, MA, USA)

### Transcriptome (RNA-seq)

Total RNA was extracted from the four cell lines, melan-a, 4C, 4C11- and 4C11+, in triplicate using TRIzol™ Reagent, and the protocol was carried out according to the manufacturer’s manual (Thermo Fisher Scientific, Waltham, MA, USA). Libraries were prepared using the Illumina TruSeqTM Stranded Total RNA Library Prep Kit with Ribo-Zero Gold for ribosomal RNA depletion (cat. #RS-122–2001, Illumina Inc., San Diego, CA, USA), using 2 μg of total RNA, according to the manufacturer’s manual. The cDNA libraries were quantified using the KAPA quantification kit (cat. # KK4835, Roche Sequencing Solutions, Pleasanton, CA, USA) and sequenced by pair-end (2 × 100 nt) on a HiSeq 1500 platform (Rapid SBS Kit v2 - 200 Cycle, Illumina Inc., San Diego, CA, USA). Raw and processed RNA-Seq data are available at the NCBI-GEO repository under accession GSE149884. Transcriptome analysis was performed by the BIDMC Precision RNA Medicine Core (under Dr. Frank Slack at Harvard Medical School). Raw sequencing reads were quality-checked using FastQC (v0.11.5) [31] and data were pre-processed with Cutadapt (v2.5) [32] for adapter removal following best practices [33]. Gene expression quantification was carried out in several steps. First, trimmed reads were quasi-aligned and quantified against the GRCm38 transcriptome using Salmon [34]. Subsequently, reads were quantified against an in-house version of the GRCm38 transcriptome, enriched for novel lncRNAs transcripts from the NONCODE database [35] (v6.0; http://www.noncode.org/). Finally, a third quantification step was performed by aligning against the GRCm38 genome using STAR (v2.7.3a) [36] and quantifying reads against Ensembl v98 [37] annotated transcript loci with featureCounts (Subread 1.6.2) [38]. Differential gene expression analysis was performed using DESeq2 (v1.24.0) [39], while ClusterProfiler (v3.12.0) [40] was utilized for downstream functional investigations. Plots were generated in R using ggplot2 (v3.3.3) [41], EnhancedVolcano (v1.8.0) [38], ComplexHeatmap (v2.6.2) [42].

### Genomic localization and identification of neighboring genes

The genomic position of the differentially expressed transcripts in at least one of the signatures was identified through the online platform NONCODEv567 (v5.0; http://www.noncode.org/) [43]. The genomic positioning data were exported and formatted to cover a genomic region of ±200 kbp for each transcript and analysed on the online platform Genome Browser (University of California, Santa Cruz, CA, USA; www.genome.ucsc.edu). We used data available in the mouse assembly – Dec. 2011 (GRCm38/mm10), extracting the following information: chromosome, start position, end position, and Gene Symbol from the ‘known canonical’ table. Of all 454 genes present in these regions, 213 are part of the DEG list previously identified in Pessoa et al. [28], and of these, 181 had orthologous genes in humans, according to the GRCm39:CM001004.3 (mouse) and GRCh38.p1 (human) databases, available on the Ensembl platform (European Molecular Biology Laboratory – EMBL-EBI, Hinxton, UK; https://www.ensembl.org) [44]. The gene encoding *Dlx4os* is located at position 11:95,035,878– 95,049,873 (forward strand, ENSMUSG00000086552). Additionally, to identify lncRNAs proximal to human *DLX4*, we used UCSC Golden Path (GRCh38/hg38) with GeneCards (www.genecards.org) and GeneCaRNA (GeneCards ncRNA compendium) custom tracks.

### TCGA and LMC cohorts

In this study, we used correlation data between the expression of human orthologs of transcripts from differentially expressed genes in malignancy (DEGs in 4C, 4C11- and 4C11+ compared with melan-a), EMT (DEGs in 4C and 4C11- compared with melan-a and 4C11+) and metastasis (DEGs in 4C11+ compared with melan-a, 4C and 4C11-) signatures and the survival of melanoma patients, using data from the Leeds Melanoma Cohort (LMC) [28]. In summary, the gene expression of 703 drug-naïve primary melanomas (Leeds Melanoma Cohort, accession number EGAS00001002922) [45] was correlated with melanoma-specific survival (MSS). Patients were classified as ‘low expression’ or ‘high expression’ based on the average gene expression across all samples, using Cox proportional hazards regression, testing the correlation through three distinct analyses: unadjusted for covariates; adjusted for age, sex, and lesion location; and adjusted for age, sex, lesion location, staging according to the AJCC (American Joint Committee on Cancer), and mitotic rate. All analyses were performed using STATA v14.1 (StataCorp, College Station, TX, USA). The data from The Cancer Genome Atlas (TCGA) cohort were accessed through the online tool Gene Expression Profiling Interactive Analysis (Gepia2) (Peking University, Beijing, China;www.gepia2.cancer-pku.cn) [46]. The samples were extracted from the Skin Cutaneous Melanoma (SKCM) database, with 461 tumour samples. Overall survival rates of patients over time were visualized using Kaplan – Meier plots with cutoff values: median 50–50%, 95% confidence interval. For both cohorts, genes that showed log-rank *p* ≤ 0.05 were considered correlated with overall patient survival for this cohort (HR > 1 = worse prognosis; HR < 1 = better prognosis).

### RT-qPCR

The RNA isolated from cell cultures using TRIzol (Invitrogen, Carlsbad, CA) was used for cDNA synthesis (reverse transcriptase – Thermo Fisher Scientific, Waltham, MA, USA) according to the manufacturer’s instructions. One microgram of each RNA was used for cDNA synthesis for subsequent expression analysis by qPCR, using LuminoCt® SYBR® Green qPCR ReadyMix™ (Sigma-Aldrich, St. Louis, MO, USA). The cDNA was synthesized using random priming and OligoDT. Specific primers for each gene were used (Supplementary Table 1) at a final concentration of 0.2 μM. The relative expression of each target gene was calculated using the Livak method [47], with *β-actin* and/or *Rpl19* as the endogenous control. Representative graphs of the results were created using GraphPad Prism version 9.3.1, with statistical analysis by unpaired t-test; *p*-value ≤ 0.05.

### Subcellular fractionation

Cell samples were fractionated using the PARIS™ Kit protocol (Invitrogen), following the manufacturer’s protocol, to separate nuclear and cytoplasmic RNA and protein. Cells were trypsinized, counted, and fractions of 7.75x10^6^ cells were centrifuged. They were suspended in 500 µl of Cell Fraction Buffer, centrifuged again, and the supernatant was collected as the cytoplasmic fraction, while the pellet was the nuclear fraction. The nuclear pellet was suspended in 500 µl of ice-cold Cell Disruption Buffer. The nuclear and cytoplasmic samples were divided into two portions for RNA and protein extraction. Fractionation efficiency was confirmed by Western blotting, and the samples of interest were analysed by qPCR.

### Knockdown of Dlx4 and Dlx4os

The 4C11- melanoma cell line was transfected with 10 nM of DsiRNAs (Dicer-substrate short interfering RNAs) DsiDlx4os (1, 2 or 3), DsiDlx4, or DsiControl (scramble) from IDT (Integrated DNA Technologies, USA) using Lipofectamine RNAiMax (Invitrogen, Carlsbad, CA), according to the manufacturer’s instructions, in RPMI medium pH 6.9 without serum and antibiotics. Six hours after transfection, fetal bovine serum and antibiotics were added to restore optimal culture conditions. After 24 hours, the cells were expanded, and 48 hours after transfection, they were counted and collected for RNA extraction. A portion was used to confirm the silencing via RT-qPCR, while the remainder was used for functional experiments.

### Overexpression of Dlx4os

Melan-a cells (1x10^6^) were transfected with pcDNA3.1_DLX4os and pcDNA3.1 (Addgene, Watertown, MA, USA) via electroporation at 150 V for 10 ms, receiving 3 pulses. After 24 hours, the cells were selected by treatment with 1 mg/ml geneticin (Gibco, Carlsbad, CA, USA) for two weeks. The overexpression of *Dlx4os* was validated by RT-qPCR.

### Coexpression analysis

The Spearman correlation coefficients were calculated between the expression of *Dlx4os* and the genes using transcriptome data (RNA-seq) [28] generated elsewhere using RStudio (v2025.09.0 + 387). Direct or inverse correlations were defined as those with a Spearman correlation coefficient greater than 0.85 or less than −0.85.

### Enrichment pathways analysis

The online platform Enrich (https://www.maayanlab.cloud/Enrichr) [48] was used to identify the functions most over-represented by the co-expressed genes. The Gene Ontology database (Biological process, BP) was used to select the enriched functions. Only functions with a *p*-value ≤ 0.05 and with at least three enriched genes were considered significant.

### Western blotting

Total protein extracts were obtained from 7x10^5^ 4C11- cells previously silenced for 48 hours for *Dlx4os* using NP-40 lysis buffer (150 mM NaCl; 1.0% NP-40; 50 mM Tris-HCl, pH 8.0) containing Halt™ Protease Inhibitor Cocktail (Thermo Fisher Scientific, Waltham, MA, USA) or using the PARIS^TM^ Kit (Invitrogen, Carlsbad, CA). Proteins were separated on a 12% SDS-PAGE gel and transferred onto PVDF membranes. The membranes were then blocked with 5% skim milk in TBS for 1 hour, followed by incubation with primary antibodies specific to each protein and corresponding secondary antibodies (Supplementary Table 2) and developed using the SuperSignal West Pico Chemiluminescent Substrate kit (Pierce, Thermo Fisher Scientific, Rockford, IL, USA) with a chemiluminescence imager – UVITEC (Cambridge). The *β-actin* expression was used as an endogenous control.

### Proliferation assay

The 4C11- cells, either silenced for *Dlx4os* (siDlx4os-1 and siDlx4os-3) or transfected with the silencing control (siNC), were counted, and 8x10^4^ were plated in 24-well plates with 500 µL of RPMI medium at pH 6.9 with 5% FBS in a 24-well plate. After 48 hours, cells were trypsinized (70 µL of trypsin per well) and counted, and normalized for total volume, ensuring the same number of cells for the silenced and control groups. Data were plotted and analysed using GraphPad Prism (GraphPad Prism/GraphPad Software, Inc).

### Clonogenicity assay

Two hundred cells from the 4C11- cell line, previously silenced for 48 hours for *Dlx4os* (siDlx4os-1 and siDlx4os-3) or transfected with the silencing control (siNC), were plated in a 6 cm diameter culture plate in RPMI medium with 5% fetal bovine serum and cultured for 10 days. Colonies were fixed with 5% formaldehyde in PBS for 5 minutes and stained with 1% Toluidine blue in 1% borax for five minutes. Cells were manually counted, then solubilized in 1% SDS for one hour at 37°C for quantification of the dye in solution by spectrophotometry (Multiskan EX, Thermo Electron, USA) at 620 nm.

### Individual migration and invasion assay

Modified Boyden chambers containing polycarbonate membranes with 8 μm pores, with or without Matrigel, were used for invasion (BD Biosciences, Franklin Lakes, NJ, USA) and migration (Transwell – Corning Inc., Corning, NY, USA) assays, respectively. The lower side of the membranes was coated with laminin at 10 μg/ml for two hours at 37°C. The laminin was then removed, and RPMI medium without serum (for migration) or with serum (for invasion) was added to the bottom of the insert. 2 × 105 4C11- cells, previously silenced for 48 hours for Dlx4os (siDlx4os-1 and siDlx4os-2) or transfected with the silencing control (siNC), were added to the upper chamber in 500 μl of serum-free RPMI and kept in a CO2 incubator for 18 or 60 hours, respectively. Cells were fixed with 4% paraformaldehyde in PBS for 15 minutes and stained with 1% Toluidine blue in 1% sodium tetraborate (borax) for five minutes. Cells were photographed under a microscope with a 20x objective (Evos Floid – Thermo Fisher Scientific, Waltham, MA, USA) and subsequently solubilized in 1% SDS for 1 hour at 37°C for quantification of the dye in solution by spectrophotometry (Multiskan EX, Thermo Electron, USA) at 620 nm.

### Collective migration assay (wound healing)

Melanoma 4C11- cells, previously silenced for 48 hours for *Dlx4os* (siDlx4os-1 and siDlx4os-2) or transfected with the silencing control (siNC), were plated at 4x10^5^ cells per well in 24-well plates. Sixteen hours after plating, a uniform healing was made in the center of the well using a 10 μl pipette tip. The culture medium was removed and replaced with RPMI medium containing 5% fetal bovine serum and 2 μg/ml of mitomycin C. The scratch was photographed immediately after it was made (Time 0) and after 12, 24, and 36 hours using a microscope (L Zeiss Primo Star – ZEISS, Germany). The healing areas were quantified using ImageJ (v1.52q; NIH, Bethesda, MD, USA).

### Anoikis resistance

For the analysis of the effect of *Dlx4os* silencing on *anoikis* resistance, 1x10^4^ cells from the 4C11- cell line silenced for 48 hours for the lncRNA *Dlx4os* (siDLX4os-1 and siDLX4os-2) or transfected with the silencing control (siNC) were maintained for 96 hours in 100 mm diameter plates coated with a 1% agarose layer to inhibit cell adhesion. After this period, the cells were plated under normal culture conditions for 16 hours, and cell viability was assessed using an MTT assay.

### PMA-independent proliferation

The cell lines 4C11- silenced for *Dlx4os* (siDlx4os-1 and siDlx4os-3) or transfected with the silencing control (siNC) were cultured with or without phorbol-myristate-acetate (PMA) added to the culture medium (final concentration of 200 nM). Forty-eight hours after plating and supplementation, the cells were trypsinized (70 µL of trypsin per well) and counted, normalized for total volume. Data were plotted and analysed using GraphPad Prism (v8.0.2 GraphPad Software, Inc.).

### Tumor growth assay (in vivo)

4C11- cells (2x10^5^ in 100 μl of PBS), silenced for 48 hours for the lncRNA *Dlx4os* (siDLX4os-1 and siDLX4os-3) or transfected with the silencing control (siNC), were subcutaneously injected into the flank of 6–8 weeks-old C57BL6 female mice (*n* = 10 animals were used per group). Mice were randomly assigned to each experimental group at the time of cell injection to minimize allocation bias. Sample size was based on previous studies with similar tumour growth models, ensuring statistical power to detect differences in tumour volume. Starting from day 20, the mice were monitored every two days to assess tumour growth; investigators responsible for measuring tumour size were blinded to group allocation to minimize observer bias. The animals were euthanized when the tumours reached a maximum size of 2.0 × 2.0 cm or showed any signs of distress. Animals that did not develop tumours were euthanized after 100 days. The procedure was approved by the Animal Use Ethics Committee (CEUA) of the university and is registered under CEUA number 1,209,191,217.

### Dlx4os homology analysis

We utilized the LiftOver genome annotation tool (https://genome.ucsc.edu/cgi-bin/hgLiftOver) from the UCSC Genome Browser [49] to identify the human genomic region syntenic to the mouse *Dlx4os* locus. The following parameters were applied: Original Genome: Mouse; Original Assembly: June 2020 (GRCm39/mm39); New Genome: Human; New Assembly: December 2013 (GRCh38/hg38). Additionally, the genomic coordinates of *Dlx4os* (chr11:95,035,878– 95,049,873) were entered into the search field.

Using the online tool Rfam (v15.0, https://rfam.org) [50], a homology search was performed based on the transcript sequence of *Dlx4os*. This analysis identified 969 RNA sequences across 12 species with homology to Dlx4os, comprising 967 lncRNAs, one miRNA, and one sRNA. Of these, one sequence was found in *Homo sapiens*, 856 in *Mus musculus*, 26 in *Mus caroli*, 24 in *Mus spretus*, 17 in *Mus spicilegus*, 13 in *Mus pahari*, 12 in *Rattus norvegicus*, 11 in *Cricetulus griseus*, five in *Chinchilla lanigera*, two in *Heterocephalus glaber*, one in *Nannospalax galili*, and one in *Felis catus*.

Using the BLAST tool [51] available on the NIH website (National Center for Biotechnology Information, Bethesda, MD, USA; https://blast.ncbi.nlm.nih.gov), an initial search was performed to compare the human transcript HSALNT0242265 with the murine *Dlx4os* sequence, focusing on conserved regions. Subsequently, the *Dlx4os* transcript was divided into 15 fragments (200 bp each), and nucleotide BLAST searches were conducted to assess their homology with the human genomic sequence at chr17:49,955,138– 49,969,894.

### HSALNG0117228 expression in human melanoma cell lines

We performed an expression analysis using transcriptomic data from Tsoi et al. (2018) (GSE80829) [6]. This dataset comprises RNA-seq data from 53 human melanoma cell lines classified into four differentiation states based on gene expression profiles. Quality control of the sequencing reads was performed using FastQC (v0.11.9), followed by alignment with STAR (v2.7.10a) [36] to the human reference genome (GRCh38.p14) using a GENCODE v49 annotation (https://www.gencodegenes.org) [52] customized to include transcripts annotated in the *HSALNG0117228 locus*. Transcript counts quantification was carried out using the featureCounts function from the Rsubread (v2.20) package [53], with multi-mapping reads excluded to ensure unambiguous quantification of *HSALNG0117228*. Genes with at least one read count in any sample were retained for downstream analysis, normalized, and variance stabilizing transformation (vst) was applied using DESeq2 (v1.50.2) [54].

### Statistical analysis

Statistical analyses of the functional assays were performed using GraphPad Prism 8.0.2 and R (v4.5.1). An unpaired Student’s t-test, log-rank (Mantel-Cox) test (4J), one-way ANOVA, and two-way ANOVA were applied to compare the results of silenced or overexpressed samples with control samples. Differences were considered statistically significant when *p* < 0.05.

## Results

### Selection of lncRnas near genes with prognostic significance in melanoma

**Figure 1.**
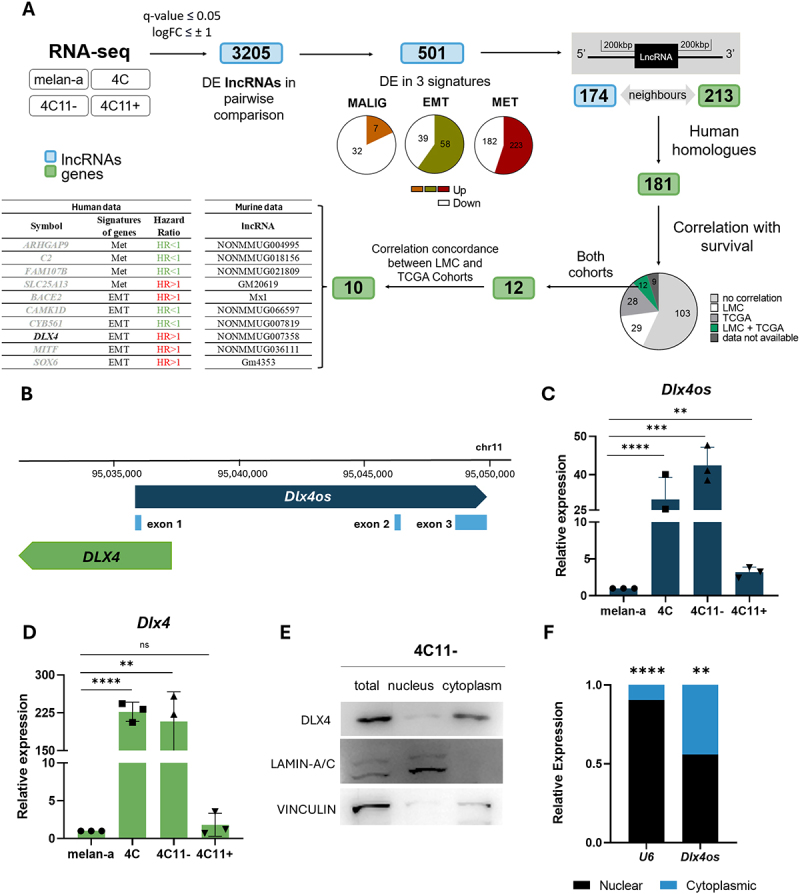
Selecting and characterizing *Dlx4os* lncRNA, a neighbor of *Dlx4* gene. (A). The pipeline used to identify differentially expressed lncRnas in melanoma, ending with the selection of our study target, *Dlx4os*. DE: differentially expressed; DEG: differentially expressed genes; LMC: Leeds melanoma Cohort; Malig: Malignancy; met: Metastasis; EMT: epithelial-mesenchymal transition. (B). Genomic region of murine *Dlx4os* and their exons, an antisense of *Dlx4*. (C-D). *Dlx4* and *Dlx4os* transcript expression levels in melan-a, 4C, 4C11- and 4C11+, measured by RT-qPCR normalized with *β-actin* relative to the expression of melan-a melanocytes. (E). The DLX4 protein is in the cytoplasm. The subcellular isolation was confirmed by Western blotting for proteins enriched in the nucleus (lamin A/C) and cytoplasm (vinculin). (F). The *Dlx4os* lncRNA is located both in the nucleus and cytoplasm, according to the qPCR quantification compared to the concentration of the *U6* RNA. The data are presented as the mean ± SD values (*n* ≥ 3). **p < 0.01; ***p < 0.001; ****p < 0.0001; ns: non significant.

To identify key lncRNAs involved in melanoma plasticity and progression, we analysed RNA-seq data of the four cell lines (melan-a, 4C, 4C11- and 4C11+) from our previously described [25] melanoma progression model (Figure 1(A)). This analysis identified 3205 differentially expressed (DE) lncRNAs across pairwise comparisons. The transcripts were grouped into three signatures associated with malignancy, epithelial-mesenchymal transition (EMT), and metastasis (Figure 1(A)).

The lncRNAs that showed differential expression in the 4C, 4C11-, and 4C11+ cell lines compared to melan-a melanocytes were classified in the ‘malignancy’ signature; whereas those differentially expressed only in the metastatic 4C11+ cell line compared to the other three cell lines were grouped in the ‘metastasis’ signature; finally, those differentially expressed in the undifferentiated and mesenchymal-like 4C and 4C11- cell lines compared to the differentiated melan-a and 4C11+ cell lines encompassed the ‘EMT’ signature. As a result, 501 lncRNAs were selected (Table S3). We initially considered *cis-regulation*, analysing the genomic region of each of the 501 lncRNAs within a distance of 200 kbp upstream and downstream, and identifying genes located in this region (neighbouring genes), selecting only those that were differentially expressed in our study model [28]. As a result, 174 lncRNAs close to 213 differentially expressed genes (DEGs) were obtained (Figure 1(A)), considering that some lncRNAs have more than one neighbouring gene within the ±200 kbp area. Subsequently, we selected only the 181 DEGs with human orthologs from the initial set of 213.

We then examined the relationship between the expression of these differentially expressed genes (DEGs) with overall survival in melanoma patients, by analysing two independent melanoma cohorts (TCGA and LMC). This identified 12 DEGs with significant prognostic value in both cohorts (*p* < 0.05). However, two of these genes showed opposite correlation trends between the cohorts and were excluded. Among the remaining DEGs with prognostic value, we identified the distal-less homeobox gene 4 (*Dlx4*) (Figure 1(B)), which has been described to be relevant in cell-fate decisions during development [55], and, therefore, we decided to further investigate the role of its lncRNA *Dlx4os* as the primary target of our study.

In our melanoma model, we observed that the expression levels of *Dlx4os* and *Dlx4* are highly expressed in 4C and 4C11- cells compared to melan-a and 4C11+ cells, which characterizes these transcripts as linked to the EMT signature (Figure 1(C,D)). These cells exhibit an undifferentiated and mesenchymal-like phenotype.

Using a subcellular fractionation kit, we separated the nuclear and cytoplasmic fractions, validated by the presence of Lamin-A/C in the nuclear and total fractions, and Vinculin in the cytoplasmic and total fractions (Figure 1(E)). By measuring the DLX4 protein level, it was possible to show its predominant localization in the cytoplasm (Figure 1(E)). Conversely, the lncRNA *Dlx4os* is present in both the nucleus and cytoplasm, as determined by qPCR and using snRNA *U6* level as a control (Figure 1(F)).

### Potential genes regulated by Dlx4os

**Figure 2.**
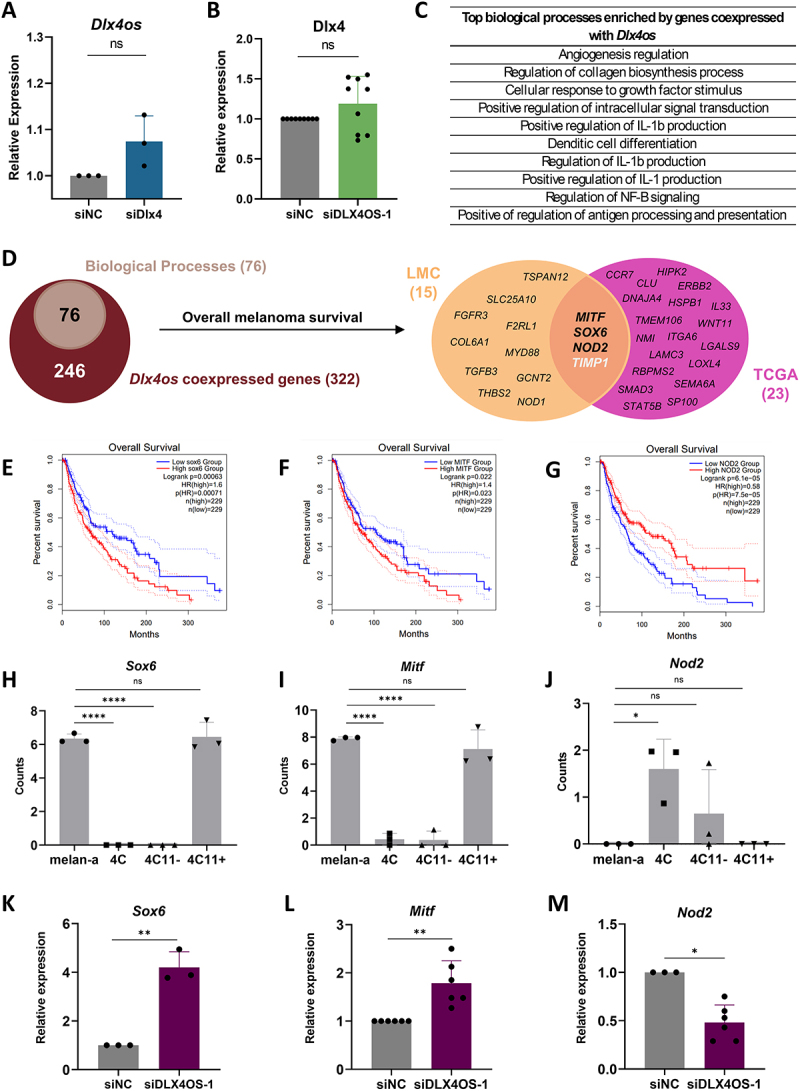
*Trans* regulation of *Dlx4os* affects phenotypic plasticity markers. (A). *Dlx4* expression in 4C11- cells silenced for *Dlx4os* (siDlx4os-1), normalized to *ß-actin* and the negative control cells (siNC). (B). *Dlx4os* expression in 4C11- cells silenced for *Dlx4* (siDlx4), normalized to *ß-actin*, and the negative control cells (siNC). (C). Top 10 biological processes enriched for genes coexpressed with *Dlx4os* using gene Ontology (GO). (D). Diagram showing how the gene selection for co-expression was performed. After selecting statistically significant hits, 76 genes involved in biological processes were chosen, and of these, 38 were associated with melanoma patient survival. Notably, MITF, NOD2, SOX6, and TIMP1 were present in both cohorts. (E-G). Overall survival of melanoma patients correlated with *Sox6*, *Mitf*, and *Nod2*, respectively, using TCGA data. The expression of *Sox6* and *Mitf* shows an inverse correlation with melanoma patient survival (HR > 1, *p*-values = 0.00063 and 0.022, respectively), whereas *Nod2* expression shows a direct correlation with survival (HR < 1, *p*-value: 7.5e-05). (H-J). *Sox6*, *Mitf*, and *Nod2* expression in the cell lines of the melanoma progression model according to previous transcriptomic data. (K-M). *Dlx4os* silencing increases the expression of *Sox6* and *Mitf* transcripts and reduces *Nod2* expression, as measured by qPCR, normalized to *ß-actin* and the negative control cells (siNC). The data are presented as the mean ± SD values (*n* ≥ 3). *p < 0.05; **p < 0.01; ****p < 0.000; ns: non significant.

To understand if there could be a relationship between *Dlx4* and *Dlx4os*, we separately silenced *Dlx4* (siDlx4) with a single siRNA, or *Dlx4os* (siDlx4os) with three different sequences of siRNA (Figures S1, A-B), and found that the expression of neither transcript is affected by the knockdown of the other gene (Figure 2(A,B)). Following knockdown of *Dlx4os*, the expression of the DLX4 protein (Figure S1) did not change either. With these results, we concluded that *Dlx4os* does not regulate *Dlx4*.

Considering that down-regulating *Dlx4os* expression did not alter the expression of the neighbouring gene *Dlx4*, we examined the transcriptome data (RNA-seq) of the melan-a, 4C, 4C11- and 4C11+ cell lines, to identify potential *trans*-regulated targets. This analysis identified genes whose expression directly or inversely correlates with the expression of *Dlx4os*, considering those with a Spearman correlation coefficient ±0.85 to account for cases of direct or inverse correlation, resulting in 322 gene hits. After that, we conducted enrichment analyses using Gene Ontology (GO), out of those, 76 genes co-expressed with *Dlx4os* were identified in 25 significantly enriched biological processes (*p*-value < 0.05) (Table S4). Highlighting the top 10 regulatory pathways were those involved in angiogenesis, regulation of collagen biosynthetic process, cellular response to growth factor stimulus, and immune correlation (Figure 2(C,D)). This analysis revealed 39 target genes, among which four had prognostic value in both cohorts (LMC and TCGA): *Mitf*, *Sox6*, *Nod2*, and *Timp1* (Figure 2(D)). *Timp1* was excluded from the analysis because the expression pattern was not consistent in the two cohorts. We then evaluated which of these genes presented a significant association with melanoma patient survival (Figure 2(E-G)).

In our melanoma model cell lines, transcriptome data revealed that all three genes (*Mitf*, *Sox6*, and *Nod2*) exhibited the same pattern of regulation as *Dlx4os*, specifically an EMT signature. *Sox6* and *Mitf* are decreased while *Nod2* is increased in 4C and 4C11- cells (Figure 2(H-J)). Additionally, this pattern aligns with the results of the coexpression analysis, where *Sox6* and *Mitf* showed an inverse correlation with *Dlx4os*, while *Nod2* was positively correlated with it. Upon silencing of *Dlx4os* (using siDlx4os-1 and siDlx4os-3), we observed an increase in *Sox6* and *Mitf* transcript levels, along with a decrease in *Nod2* expression (Figure 2(K-M); S1, C-E), confirming the findings of this correlation analysis, and showing that *Dlx4os* is required for normal expression of these genes.

The *Nod2* gene is important in melanoma development [56,57], whereas *Mitf* and *Sox6* are two master regulator genes correlated with phenotype switching, which frequently occurs due to melanoma plasticity [7,58,59]. Specifically, MITF is a key regulator of melanogenesis and cell differentiation, and its expression levels vary among tumour cells, with low MITF levels associated with increased invasiveness [5,8], being one of the most crucial genes in this process. Based on this scenario, our findings led us to investigate whether *Dlx4os* is involved in phenotype transitions.

### Dlx4os lncRNA regulates phenotype plasticity in melanoma

**Figure 3.**
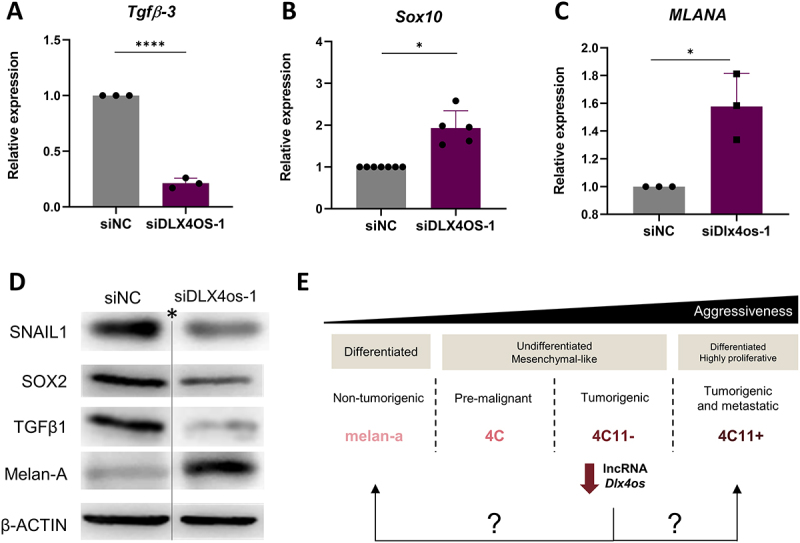
*Dlx4os* promotes phenotype switching in melanoma cells. (A-C). *Dlx4os* silencing decreases, respectively, the expression of *Tfgβ-3* and increases *Sox10* and *mlana* expression, as measured by qPCR, normalized to *ß-actin* and the negative control cells (siNC). (D). Representative images of Western blotting for the expression of SNAIL1, SOX2, TGF-ß1, and MELAN-A with *Dlx4os* silencing in the 4C11- cell line. *ß-actin* was used as an endogenous control. (E). The silencing of *Dlx4os* directs the undifferentiated and mesenchymal-like 4C11- melanoma cells toward a differentiated state, like that observed in melan-a melanocytes and 4C11+ melanoma cells. The data are presented as the mean ± SD values (*n* ≥ 3). *p < 0.05; ****p < 0.0001 *original image available on suplemmentary Figure S1J.

The different states of melanoma can be distinguished by the expression of specific markers present in each state. Therefore, to begin testing our hypothesis that *Dlx4os* is an important lncRNA regulating melanoma plasticity, we analysed the transcript (Figure 3(A-C)) and protein expression of specific biomarkers of phenotypic states (Figure 3(D); S1, F-H) following *Dlx4os* knockdown.

We observed that *Dlx4os* knockdown leads to decreased expression of important biomarkers of mesenchymal-like, undifferentiated state as SNAIL1, SOX2, TGFβ-1 (Figure 3(D); S1, G-H), and *Tgf-b3* (Figure 3(A); S1F). Additionally, there is an increase in *MLANA*, *Sox10* (Figure 3(B,C), respectively), and *Mitf* (Figure 2(L)) expression, which are known molecular markers in the characterization of the differentiated state observed in advanced melanomas. These data led us to conclude that *Dlx4os* silencing promotes a phenotype switch in melanoma cells from an undifferentiated, mesenchymal-like to a more differentiated state.

### Dlx4os lncRNA knockdown confers a less malignant phenotype in 4C11-melanoma cells

The murine lines used here recapitulate the phenotypes observed in human melanoma lines [28]. It is known that the 4C and 4C11- cells exhibit a mesenchymal-like phenotype, being poorly proliferative and undifferentiated, while melan-a and 4C11+ cells display a well-differentiated phenotype, with 4C11+ cells being highly proliferative and metastatic (Figure 3(E)). Thus, to test if the cells are transiting to a differentiated state, with less aggressiveness (like melan-a cell line), or to a differentiated and aggressive (like 4C11+ cell line) (Figure 3(E)), we evaluated biological characteristics that could differ between the melan-a melanocytes and 4C11+ melanoma cells.

**Figure 4.**
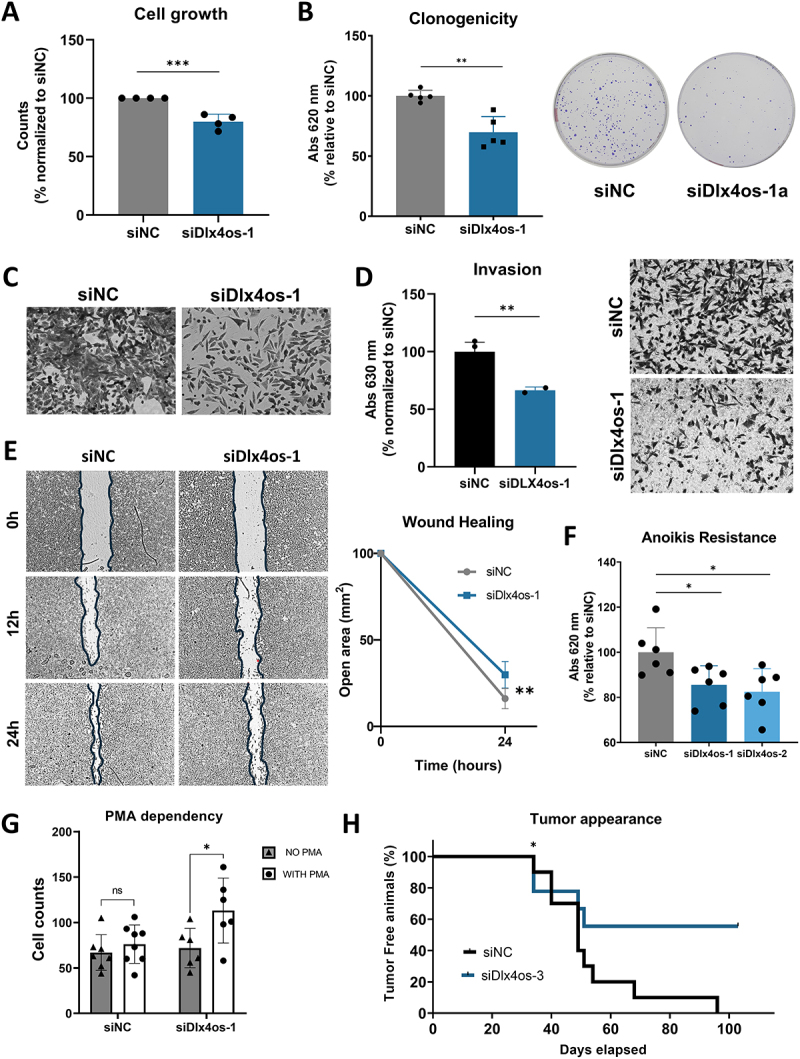
*Dlx4os* knockdown promotes a less malignant phenotype in melanoma cells. (A). Cell proliferation was assessed by counting viable cells after 48 hours of silencing, performed manually using a Neubauer chamber. Percentage relative to the control (siNC). (B). Quantification of colonies estimated by the absorbance of the solution after lysing cells stained with Toluidine blue using ImageJ 1.52q software (left), and photographic representation of colonies stained with Toluidine blue (right). (C). Cell individual migration after 4 hours, in laminin-coated inserts, in serum-free medium (10 μg/ml; images at 20X magnification (D). Quantification of Toluidine blue dye absorbance shown in the graph (left) and image of invasion after 64 hours, in laminin-coated inserts, in RPMI medium supplemented with 5% serum (10 μg/ml; images at 20X magnification) (right). (E). Representative image of scratch wounds (10X) at 0, 12, and 24 hours analyzed using ImageJ 1.52q software (left) and quantification of the open area (mm^2^) 24 hours after wounding (right). (F). Count of *anoikis*-resistant cells after *Dlx4os* silencing relative to control. (G). Cell culture performed in the absence (NO PMA – gray) or presence (WITH PMA – white) of PMA in 4C11. (H). graph of *in vivo* tumor appearance for *Dlx4os* silencing versus control (*n* = 10 per group). The data are presented as mean ± SD values (*n* ≥ 3). *p < 0.05; **p < 0.01; ***p < 0.001.

To investigate the biological function of *Dlx4os* in melanoma, we first analysed the proliferation rate of 4C11- cells 48 hours after being silenced for *Dlx4os*, and we observed a reduced number of *Dlx4os* knocked down cells compared to control (Figure 4(A); S2A). This result was confirmed by the clonogenic assay, where we observed fewer colonies after *Dlx4os* was knocked down (Figure 4(B); S2B).

Furthermore, the knockdown of *Dlx4os* significantly altered the individual (Figure 4(C); S2C) and collective (Figure 4(E) ; S2D), and invasion capacities of 4C11- cells (Figure 4(D); S2E). Additionally, the *anoikis* resistance assay revealed that the cells silenced for *Dlx4os* are less resistant to cell blockade death (Figure 4(F)). Together, these findings suggest that silencing the lncRNA *Dlx4os* reduces the aggressiveness of melanoma cells, guiding the undifferentiated, mesenchymal-like 4C11- melanoma cells towards a state like non-transformed cells (as melan-a cells) rather than an aggressive state (such as 4C11+ melanoma cells). A key characteristic of melanocytes is their dependency on PMA for proliferation and survival [60]. Given this characteristic, we confirmed that *Dlx4os-*silenced 4C11- cells display increased PMA dependency for proliferation compared to the control (siNC) (Figures 4(G); S2F).

Finally, to evaluate the *in vivo* effect of the *Dlx4os* knockdown on tumour growth, *Dlx4os* knockdown 4C11- cells were subcutaneously injected into mice. Melanoma cells expressing decreased levels of *Dlx4os* exhibited a longer latency time for tumour appearance compared to the control group (siNC) (Figures 4(H); S2G). This result aligns with *in vitro* findings.

**Figure 5.**
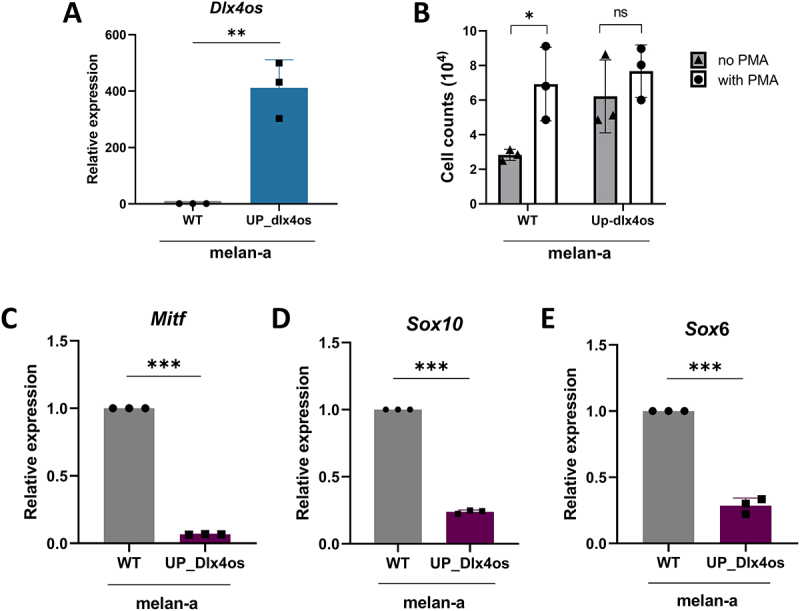
The overexpression of *Dlx4os* in melan-a leads to a mesenchymal-like phenotype. (A). The expression of *Dlx4os* was evaluated in melan-a cells, with or without overexpression, using RT-qPCR relative to negative control cells (siNC), with *Rpl19* as the reference gene. (B). Cell culture performed in the absence (NO PMA – gray) or presence (WITH PMA – white) of PMA in 4C11. (C-E). *Dlx4os* overexpression decreases the expression of *Mitf*, S*ox10*, and *Sox6*, as measured by qPCR, normalized to *ß-actin* and the negative control cells (siNC). The data are presented as mean ± SD values (*n* ≥ 3). *p-value < 0.05; **p-value < 0.01; ***p-value < 0.001.

In accordance with the results from silencing *Dlx4os* in 4C11+ cells, when *Dlx4os* was overexpressed in the melan-a cell line (Figure 5(A)), the cells lost their dependency on PMA for proliferation, displaying similar proliferation rates regardless of the presence or absence of PMA (Figure 5(B)). Additionally, the levels of Mitf, Sox10, and Sox6 (Figure 5(C-E)) decreased, confirming that the lncRNA *Dlx4os* drives an undifferentiated, mesenchymal-like phenotype and contributes to melanocyte malignant transformation.

### Identification of a potential human ortholog of Dlx4os

**Figure 6.**
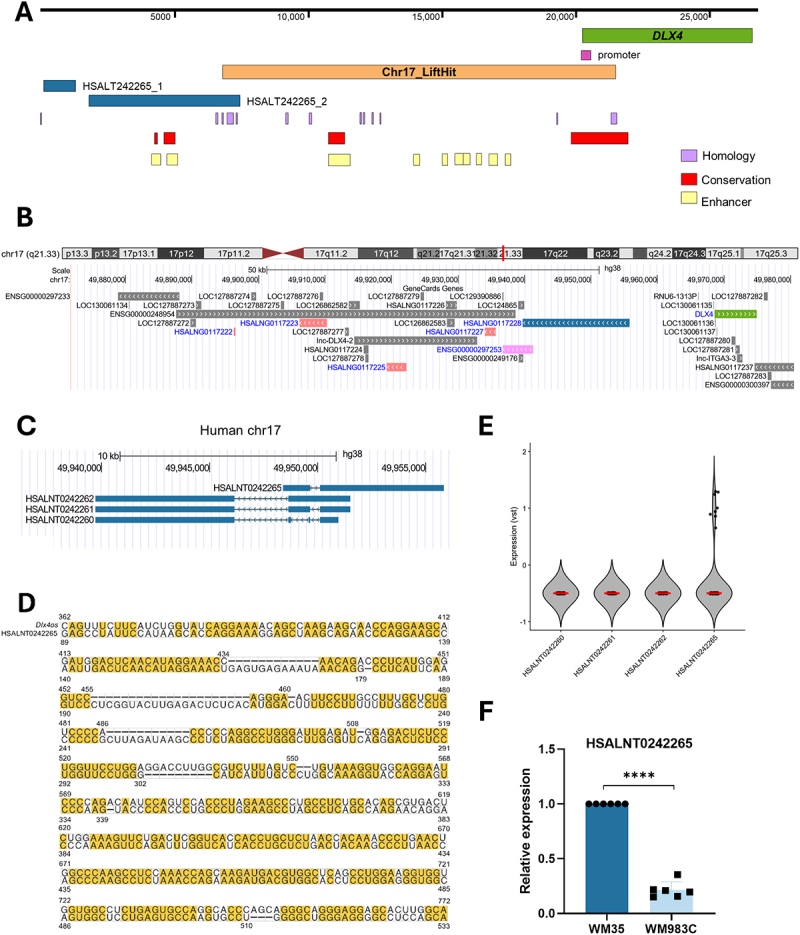
Search for homologue *Dlx4os* in humans. (A). Graphical representation of the human chromosomal region with the potential to transcribe a non-coding RNA orthologue to murine *Dlx4os*. The human genome’s chromosomal region chr17:49,955,138– 49,969,894 (represented by the black line, 14,756 bp) exhibits several features suggesting the possible transcription of a long non-coding RNA not yet annotated. In orange, the chromosomal region resulting from the lift-over performed with the murine *Dlx4os* transcript sequence. In purple, chromosomal regions with sequence homology between murine *Dlx4os* and the human genome, identified by nucleotide blast search, including two homologous regions overlapping the *Dlx4os* lift-over region and HSALNT0242265 (homology selected). In red, regions identified as highly conserved across species. In light yellow, enhancer regions. (B). Genomic region chr17:49,885,135– 49,993,776 of the human genome (GRCh38/hg38). *DLX4* (green), *HSALNG0117228* (blue), *ENSG00000297253* (pink), *HSALNG0117227* (red), *HSALNG0117225* (red), *HSALNG0117223* (red), and *HSALNG0117222* (red). Figure generated using UCSC genome Browser and UCSC Golden Path with GeneCards custom tracks. (C). Representative image showing the transcripts encoded in the HSALNG0117228 locus and their chromosomal position. Figure generated using UCSC genome Browser and UCSC Golden Path with GeneCards custom tracks. (D). Sequence alignment of HSALNT0242265 and *Dlx4os*. (E). Variance-stabilized (VST)-normalized absolute expression of HSALNT0242260, HSALNT0242261, HSALNT0242262, HSALNT0242265 in Tsoi et al. (2018) (GSE80829) data. The red line represents the median. (F). HSALNT0242265 transcript expression was measured using cDNA of different human melanoma cell lines by qPCR. Normalized to *GAPDH* and the negative control. The data are presented as the mean ± SD values (*n* = 6). ****p < 0.0001.

To explore the clinical relevance of Dlx4os, we performed a comprehensive analysis to search for a human ortholog of this lncRNA. No counterpart has been reported in the literature or detected through BLAST searches. Therefore, we used complementary strategies to investigate the possible existence of a human ortholog. We initially employed a synteny-based strategy to convert genomic coordinates between the reference mouse and human assemblies, enabling the exploration of orthologous regions that were not previously annotated. Specifically, we used the UCSC LiftOver tool to map the corresponding region of murine *Dlx4os* in the human genome (GRCh38/hg38). This analysis revealed a region on chromosome 17 (chr17:49,955,138– 49,969,894), much of which remains unannotated for known genes (Figure 6(A)). Notably, this region is near the protein-coding gene *DLX4* and contains multiple *cis*-regulatory elements. It is highly conserved across several species and shows a high density of enhancer-associated regions, suggesting that it may be transcriptionally active.

Following this analysis, we examined the syntenic region surrounding human *DLX4*, the neighbouring gene of *Dlx4os* in mice, using UCSC Golden Path with GeneCards custom tracks. Analysis of the *DLX4* genomic region revealed six predicted lncRNA loci positioned on the opposite strand, upstream of *DLX4*, in agreement with the genomic location and orientation of *Dlx4os* in the murine genome. These lncRNAs include *HSALNG0117228* (chr17:49,939,724– 49,955,818), *ENSG00000297253* (chr17:49,936,795– 49,941,281), *HSALNG0117227* (chr17:49,934,028– 49,935,702), *HSALNG0117225* (chr17:49,919,314– 49,922,224), *HSALNG0117223* (chr17:49,906,193– 49,910,268), and *HSALNG0117222* (chr17:49,896,242– 49,896,523) (Figure 6(B)). In this context, *HSALNG0117228*, the closest predicted lncRNA on the opposite strand to *DLX4*, encodes four transcripts: HSALNT0242262 (chr17:49,939,724– 49,951,504), HSALNT0242261 (chr17:49,939,724– 49,951,504), HSALNT0242260 (chr17:49,939,724– 49,950,949), and HSALNT0242265 (chr17:49,948,397– 49,955,818) (Figure 6(C)).

To evaluate sequence homology between the murine lncRNA *Dlx4os* and the human genomic region identified via LiftOver, we divided the 1664 bp *Dlx4os* transcript into overlapping 200 bp fragments. Each fragment was subjected to nucleotide BLAST analysis against the human sequence to ensure full coverage. This approach yielded 16 fragments and identified 13 homologous regions (Figure 6(A), Figure S3A). Considering all 13 regions, two alignments overlapped the syntenic region identified via liftOver of murine Dlx4os and HSALNT0242265: one between positions 263–503 of HSALNT0242265 and 492–739 of *Dlx4os* (73% identity, 6% gaps, E-value < 0.0001), and another between positions 108–160 and 381–433 (83% identity, 0% gaps, E-value < 0.001) (Homology selection in Figure 6(A)). No other transcripts or neighbouring genes displayed significant sequence similarity to *Dlx4os*. Notably, the conserved sequence between *Dlx4os* and HSALNT0242265 was located at the beginning of the HSALNT0242265 sequence, likely corresponding to its promoter region. Promoter regions in lncRNAs are known to be more conserved than those in protein-coding genes, maintaining their regulatory function [61]. Inspection of putative regulatory elements in the genomic sequence spanning the HSALNT0242265 locus showed the presence of a CTCF binding site within its first exon that is strongly supported by ENCODE ChIP-seq data from different cell types, including keratinocytes (Figure S3B). This region is also decorated by enhancer-associated H3K4me1 marks from different cell lines. No significant promoter-associated H3K4me3 marks were observed at the putative TSS. Of note, the ENCODE ChIP-seq data do not include melanoma cells (Figure S3B).

Additionally, we utilized the Rfam database (v15.0) to explore homologs of the murine *Dlx4os*. This analysis identified a single human RNA sequence, HSALNT0242265 as a putative homolog (E-value < 0.001), with 6% of HSALNT0242265 and 25% of *Dlx4os* sharing sequence similarity (63% identity, 13% gaps, 24,7% of query coverage, 6,4% of target coverage, E-value < 0.0001) (Figure 6(D)), strengthening our hypothesis regarding homology conservation between the murine *Dlx4os* locus and the syntenic region of the human genome. The HSALNT0242265 transcript consists of two exons, located at chr17:49,948,397– 49,949,649 (1,252 bp) and chr17:49,950,116– 49,955,818 (5,702 bp), and is the closest transcript to the *DLX4* locus, separated by 12,754 bp. In contrast, *ENSG00000297253*, the second closest predicted lncRNA, encodes a single transcript, ENST00000746578.1, with three exons located at chr17:49,936,795– 49,936,883, chr17:49,939,238– 49,939,384, and chr17:49,940,750– 49,941,281. Notably, the ENST00000746578.1 transcript does not overlap with the HSALNT0242265 transcript.

To assess the expression of *HSALNG0117228* transcripts, we analysed the RNA-seq dataset published by Tsoi et al. (GSE80829) [6], which includes transcriptomic profiles from 58 samples derived from 53 human melanoma cell lines representing different phenotypic states. HSALNT0242265 exhibited detectable expression; however, most samples showed low or undetectable levels. The remaining transcripts displayed no detectable expression (Figure 6(E)). To further document whether HSALNT0242265 is transcribed in human melanoma cells, we performed qPCR analysis on RNA extracted from human melanoma cell lines, using WM35 as a model of primary melanoma and WM983C as a model of metastatic melanoma. The transcript was detected, albeit at low levels, in primary melanoma cells but not in the metastatic cell line, consistent with observations in the murine model (Figure 6(F)). These results provide evidence that the region spanning chr17:49,948,397– 49,955,818 is transcriptionally active when compared with the negative control (Figure 3SC).

Taken together, our results indicate that the lncRNA *Dlx4os* is conserved between mice and humans and is expressed in human melanoma cells and tissues, corresponding at least in part to HSALNT0242265.

## Discussion

We observed a strong association between Dlx4os expression and key regulators of melanoma cell plasticity, particularly MITF and SOX6. *Dlx4os* is highly expressed in undifferentiated, mesenchymal-like melanoma cells and its downregulation shifts these cells towards a less aggressive, melanocyte-like state. Notably, this effect occurs independently of changes in its sense counterpart DLX4, indicating that *Dlx4os* likely exerts its function through trans-acting regulatory mechanisms. MITF, a master regulator of melanocyte development and differentiation, controls the transcription of numerous genes and also melanocyte growth, survival, and differentiation [62]. Furthermore, it is also known to define melanoma cell states, with low levels promoting invasiveness and high levels favouring differentiation and proliferation [58,63,64]. In parallel, SOX6 was previously linked to a more aggressive/dedifferentiated phenotype in melanoma [65]. High levels of SOX6 were recently described to be involved with an intermediary cell state [7], in which a symbiotic mixture of cells in either melanocytic or mesenchymal-like states would result in increased migration, invasion, and drug resistance capacity.

Mechanistically, *Dlx4os* might act in trans by modulating chromatin accessibility and transcriptional networks at distant genomic loci. Possible mechanisms include the recruitment of epigenetic modifiers (such as histone methyltransferases or chromatin remodellers) [66] to *Mitf* and *Sox6* regulatory regions, interaction with transcription factors or co-regulators to fine-tune their activity, or functioning as a molecular scaffold [8] to coordinate multi-protein complexes that sustain the dedifferentiated state. Additionally, *Dlx4os* may regulate post-transcriptional networks by acting as a competing endogenous RNA (ceRNA) that sequesters microRNAs [12] targeting *Mitf* or *Sox6* transcripts, thereby indirectly controlling their expression. Together, these findings support a model in which *Dlx4os* operates as a trans-acting regulator of melanoma cell plasticity, promoting a dedifferentiated and more aggressive phenotype through coordinated control of *Mitf*- and *Sox6*-dependent transcriptional programmes.

Our data showed that *Dlx4os* knockdown increased MITF and SOX6 expression, suggesting that *Dlx4os* functions as a negative regulator of differentiation. These findings are consistent with its expression pattern in *in vitro* melanoma models, as we demonstrated here that *Dlx4os* is strongly associated with the epithelial-to-mesenchymal transition (EMT) signature in melanoma, especially in undifferentiated, mesenchymal-like cells (4C and 4C11- cell lines). Nonetheless, it is worth mentioning that although 4C11+ cells express higher levels of *Dlx4os* than melan-a cells, an abrupt decrease is observed in 4C11+ compared to 4C11- cells, probably due to molecular alterations that confer high proliferative and metastasis capacity.

Additionally, silencing *Dlx4os* redirected these cells towards a more differentiated phenotype, as evidenced by increased expression of differentiation markers (e.g., MLANA, SOX10, and MITF) and decreased expression of mesenchymal-like markers (e.g., SNAIL, SOX2, and TGF-ß), as shown in the literature [2,6,7]. This suggests that *Dlx4os* functions as a driver of an undifferentiated, invasive phenotype. This phenotypic switch reflects a reversal of the mesenchymal-like state, aligning the cells closer to the differentiated phenotype, observed in melan-a and 4C11+ cells. We also found that *Dlx4os* knockdown not only reduced cell proliferation and colony formation but also significantly impaired migration, invasion, and *anoikis* resistance – key features of aggressive melanoma cells. Importantly, the increased dependency on PMA for proliferation in *Dlx4os*-silenced 4C11− cells, along with the decreased dependency on PMA in *Dlx4os*-overexpressing melan-a cells, further underscores their shift towards a more melanocyte-like phenotype, resembling non-transformed cells. The *in vivo* results corroborated these findings, with *Dlx4os* knockdown extending the latency time for tumour formation, further emphasizing its role in sustaining the transformed phenotype.

There is no work in the literature investigating the role of *Dlx4os* in cancer, particularly in melanoma. Another lncRNA *H19*, was described to inhibit cell growth, invasion, and migration as well as to differentially regulate the expression of epithelial-mesenchymal transition (EMT)-related gene expression and reverse EMT in melanoma cell lines [67]. Similar to *Dlx4os*, knockdown of *H19* suppressed *in vivo* tumour growth and modulated the expression of EMT-related genes. Our data suggest that *Dlx4os* is another lncRNA that acts as an important regulator that modulates melanoma plasticity, tending towards an undifferentiated, mesenchymal-like phenotype, which is crucial for tumour progression, contributing to maintaining a malignant phenotype.

One of the main challenges in studying lncRNAs is their generally low evolutionary conservation at the primary sequence level. For example, whole-genome alignments revealed that approximately 20% of human lncRNAs have homologs in mice, compared to around 99% of human protein-coding genes that have a mouse homolog [68–70]. When analysing the lncRNA *Dlx4os*, we observed that its ortholog had not been previously described.

It is well-established, however, that the conservation of lncRNAs is a complex phenomenon [71]. Conservation can be analysed across multiple dimensions, including sequence similarity, transcription and processing status, positional conservation, and structural conservation [70,71]. Therefore, in our search for conservation, we not only based our approach on sequence similarity but also considered the syntenic conservation of the chromosomal region spanning *Dlx4os*. One of the characteristics that reinforces our theory is that we identified that the beginning of the sequence of this transcript, where the promoter region is located, contains a large region of sequence conservation relative to *Dlx4os*, which aligns with the literature, as current knowledge suggests a high degree of conservation in promoter regions [61].

By combining multiple strategies, including database searches (Rfam), LiftOver genome alignment, and nucleotide BLAST analysis, we identified the HSALNT0242265 transcript, from the *HSALNG0117228* locus, as a strong candidate for the human ortholog of the murine lncRNA *Dlx4os*. Furthermore, RT-PCR amplification of this transcript in different human melanoma cell lines confirmed that HSALNT0242265 is not only a potential ortholog but is also transcribed in these cells. Moreover, we reanalysed publicly available bulk RNA-seq dataset [6] and obtained additional evidence that HSALNT0242265 is expressed in human melanoma cell lines. A common feature of many lncRNAs is the absence of a poly(A) tail [72], and the detection of these transcripts often require specific enrichment protocols during RNA-seq library preparation. The available melanoma RNAseq datasets were generated from poly(A)-enriched libraries [72], which could explain the low expression levels of *HSALNG0117228* transcripts. Our study is the initial evidence of the corresponding transcript of *Dlx4os* in humans and fills an important gap in our understanding of lncRNA conservation across species. Further studies are needed to investigate the regulatory elements, cellular localization, and functional relevance of HSALNT0242265 in human melanoma and other contexts.

## Conclusion

Our study highlights the crucial role of the lncRNA *Dlx4os* in a model of melanoma progression. The discovery of a lncRNA linked to phenotypic plasticity is particularly important because this trait could be controlled to modulate melanoma’s response to specific treatments. Additionally, *Dlx4os* could be an important biomarker of melanoma in its initial stages. Moreover, this study is the first to identify a possible human ortholog for the murine lncRNA *Dlx4os*, a transcript previously lacking any annotated counterpart in humans. This finding is an essential step towards translational applications, enabling further exploration of its role in human melanoma and its potential as a therapeutic target.

## Supplementary Material

Table S4.docx

Table S3.docx

Figure S1.TIF

Figure S2.TIF

Supplementary Material.docx

Table S2.docx

Table S1.docx

## Data access statement

All data generated or analysed during this study are included in this published article and its supplementary information files. The datasets generated and analysed during the current study are available in the Zenodo repository (DOI: https://doi.org/10.5281/zenodo.18806375) and in the Gene Expression Omnibus (GEO) database under accession number GSE149884 for RNA-seq data. Further information is available from the corresponding author upon reasonable request.
