# Supplementary material for: LncRNA Dlx4os drives malignant transformation and phenotype switching in melanoma
Source: Epigenetics. 2026 Mar 19;21(1):2641924. doi: 10.1080/15592294.2026.2641924 (PMC13003849; doi:10.1080/15592294.2026.2641924)
Supplement: Table S3.docx [file KEPI_A_2641924_SM4556.docx]

| chr | | TRANSCRIPT NAME | LncRNA Class | SIGNATURE | DIRECTION |
| --- | --- | --- | --- | --- | --- |
| chr | 1 | NONMMUG048626 | Linc | EMT | DOWN |
| chr | 1 | NONMMUG047617 | Linc | EMT | DOWN |
| chr | 1 | NONMMUG000567 | Linc | EMT | UP |
| chr | 1 | NONMMUG000991 | Exonic | EMT | UP |
| chr | 1 | Gm29100 | lncRNA | EMT | UP |
| chr | 1 | NONMMUG001361 | Sense no Exonic | EMT | UP |
| chr | 1 | NONMMUG001822 | Linc | EMT | UP |
| chr | 1 | NONMMUG002548 | Linc | MALIG | DOWN |
| chr | 1 | NONMMUG047671 | Linc | MALIG | DOWN |
| chr | 1 | NONMMUG046632 | Sense no Exonic | METAS | DOWN |
| chr | 1 | NONMMUG047121 | Sense no Exonic | METAS | DOWN |
| chr | 1 | NONMMUG047199 | Linc | METAS | DOWN |
| chr | 1 | NONMMUG087678 | Linc | METAS | DOWN |
| chr | 1 | NONMMUG001887 | Linc | METAS | DOWN |
| chr | 1 | NONMMUG002471 | Linc | METAS | DOWN |
| chr | 1 | NONMMUG049435 | Linc | METAS | DOWN |
| chr | 1 | NONMMUG087584 | Linc | METAS | UP |
| chr | 1 | NONMMUG000420 | Exonic | METAS | UP |
| chr | 1 | NONMMUG048348 | Antisense | METAS | UP |
| chr | 1 | NONMMUG046996 | Linc | METAS | UP |
| chr | 1 | NONMMUG001327 | Antisense | METAS | UP |
| chr | 1 | NONMMUG001466 | Linc | METAS | UP |
| chr | 1 | NONMMUG001638 | Linc | METAS | UP |
| chr | 1 | NONMMUG001660 | Linc | METAS | UP |
| chr | 1 | NONMMUG047457 | Linc | METAS | UP |
| chr | 1 | NONMMUG047536 | Antisense | METAS | UP |
| chr | 1 | NONMMUG002594 | Exonic | METAS | UP |
| chr | 2 | NONMMUG066689 | Linc | EMT | DOWN |
| chr | 2 | NONMMUG092122 | Exonic | EMT | DOWN |
| chr | 2 | NONMMUG024494 | Sense no Exonic | EMT | DOWN |
| chr | 2 | NONMMUG068874 | Linc | EMT | DOWN |
| chr | 2 | NONMMUG025550 | Antisense | EMT | DOWN |
| chr | 2 | NONMMUG025564 | Antisense | EMT | DOWN |
| chr | 2 | NONMMUG022340 | Linc | EMT | UP |
| chr | 2 | NONMMUG022562 | Linc | EMT | UP |
| chr | 2 | NONMMUG022684 | Linc | EMT | UP |
| chr | 2 | NONMMUG023124 | Antisense | EMT | UP |
| chr | 2 | NONMMUG023721 | Sense no Exonic | EMT | UP |
| chr | 2 | NONMMUG023848 | Antisense | EMT | UP |
| chr | 2 | NONMMUG025670 | Linc | MALIG | UP |
| chr | 2 | NONMMUG021809 | Sense no Exonic | METAS | DOWN |
| chr | 2 | NONMMUG066597 | Antisense | METAS | DOWN |
| chr | 2 | NONMMUG022390 | Linc | METAS | DOWN |
| chr | 2 | NONMMUG022413 | Antisense | METAS | DOWN |
| chr | 2 | NONMMUG022518 | Linc | METAS | DOWN |
| chr | 2 | NONMMUG023099 | Exonic | METAS | DOWN |
| chr | 2 | NONMMUG023476 | Linc | METAS | DOWN |
| chr | 2 | NONMMUG023935 | Linc | METAS | DOWN |
| chr | 2 | NONMMUG024175 | Sense no Exonic | METAS | DOWN |
| chr | 2 | NONMMUG024302 | Linc | METAS | DOWN |
| chr | 2 | NONMMUG024452 | Exonic | METAS | DOWN |
| chr | 2 | NONMMUG024479 | Linc | METAS | DOWN |
| chr | 2 | NONMMUG024539 | Exonic | METAS | DOWN |
| chr | 2 | NONMMUG024578 | Sense no Exonic | METAS | DOWN |
| chr | 2 | NONMMUG025090 | Linc | METAS | DOWN |
| chr | 2 | NONMMUG025486 | Exonic | METAS | DOWN |
| chr | 2 | NONMMUG025574 | Linc | METAS | DOWN |
| chr | 2 | NONMMUG068887 | Linc | METAS | DOWN |
| chr | 2 | NONMMUG025748 | Linc | METAS | DOWN |
| chr | 2 | NONMMUG092620 | Linc | METAS | DOWN |
| chr | 2 | NONMMUG025749 | Linc | METAS | DOWN |
| chr | 2 | NONMMUG025750 | Antisense | METAS | DOWN |
| chr | 2 | NONMMUG025980 | Linc | METAS | DOWN |
| chr | 2 | NONMMUG022181 | Linc | METAS | UP |
| chr | 2 | NONMMUG022255 | Exonic | METAS | UP |
| chr | 2 | NONMMUG022268 | Antisense | METAS | UP |
| chr | 2 | NONMMUG022454 | Exonic | METAS | UP |
| chr | 2 | NONMMUG023068 | Sense no Exonic | METAS | UP |
| chr | 2 | NONMMUG023451 | Antisense | METAS | UP |
| chr | 2 | NONMMUG023453 | Sense no Exonic | METAS | UP |
| chr | 2 | NONMMUG023561 | Exonic | METAS | UP |
| chr | 2 | NONMMUG023593 | Linc | METAS | UP |
| chr | 2 | NONMMUG067176 | Linc | METAS | UP |
| chr | 2 | NONMMUG023633 | Linc | METAS | UP |
| chr | 2 | NONMMUG023937 | Linc | METAS | UP |
| chr | 2 | NONMMUG024232 | Sense no Exonic | METAS | UP |
| chr | 2 | NONMMUG024278 | Linc | METAS | UP |
| chr | 2 | NONMMUG024510 | Exonic | METAS | UP |
| chr | 2 | NONMMUG024511 | Exonic | METAS | UP |
| chr | 2 | NONMMUG024712 | Antisense | METAS | UP |
| chr | 2 | NONMMUG025066 | Linc | METAS | UP |
| chr | 2 | NONMMUG025251 | Sense no Exonic | METAS | UP |
| chr | 2 | NONMMUG025546 | Linc | METAS | UP |
| chr | 2 | NONMMUG025703 | Linc | METAS | UP |
| chr | 3 | NONMMUG027430 | Exonic | EMT | DOWN |
| chr | 3 | NONMMUG070920 | Antisense | METAS | DOWN |
| chr | 3 | NONMMUG026453 | Linc | METAS | DOWN |
| chr | 3 | NONMMUG027079 | Exonic | METAS | DOWN |
| chr | 3 | NONMMUG071245 | Linc | METAS | DOWN |
| chr | 3 | NONMMUG027443 | Linc | METAS | DOWN |
| chr | 3 | NONMMUG028241 | Linc | METAS | DOWN |
| chr | 3 | NONMMUG026551 | Linc | METAS | UP |
| chr | 3 | NONMMUG026552 | Linc | METAS | UP |
| chr | 3 | NONMMUG027012 | Antisense | METAS | UP |
| chr | 3 | NONMMUG027093 | Linc | METAS | UP |
| chr | 3 | Gm15417 | lncRNA | METAS | UP |
| chr | 3 | NONMMUG027312 | Antisense | METAS | UP |
| chr | 3 | NONMMUG092958 | Linc | METAS | UP |
| chr | 3 | NONMMUG071247 | Linc | METAS | UP |
| chr | 3 | Gm5546 | lncRNA | METAS | UP |
| chr | 3 | NONMMUG070111 | Sense no Exonic | METAS | UP |
| chr | 4 | NONMMUG028483 | Exonic | EMT | DOWN |
|  |  |  |  | METAS | UP |
| chr | 4 | NONMMUG028975 | Antisense | EMT | DOWN |
| chr | 4 | NONMMUG029165 | Exonic | EMT | DOWN |
| chr | 4 | NONMMUG072477 | Linc | EMT | DOWN |
| chr | 4 | NONMMUG029064 | Linc | MALIG | DOWN |
| chr | 4 | NONMMUG029562 | Linc | MALIG | DOWN |
| chr | 4 | Gm49890 | lncRNA | MALIG | DOWN |
| chr | 4 | NONMMUG029945 | Linc | MALIG | DOWN |
| chr | 4 | NONMMUG029954 | Sense no Exonic | MALIG | DOWN |
| chr | 4 | NONMMUG029955 | Antisense | MALIG | DOWN |
| chr | 4 | NONMMUG072825 | Linc | METAS | DOWN |
| chr | 4 | NONMMUG028686 | Linc | METAS | DOWN |
| chr | 4 | NONMMUG028863 | Exonic | METAS | DOWN |
| chr | 4 | NONMMUG029073 | Linc | METAS | DOWN |
| chr | 4 | NONMMUG093375 | Linc | METAS | DOWN |
| chr | 4 | NONMMUG029813 | Antisense | METAS | DOWN |
| chr | 4 | NONMMUG029868 | Antisense | METAS | DOWN |
| chr | 4 | NONMMUG031169 | Linc | METAS | DOWN |
| chr | 4 | NONMMUG072694 | Linc | METAS | DOWN |
| chr | 4 | NONMMUG031291 | Linc | METAS | DOWN |
| chr | 4 | NONMMUG073887 | Linc | METAS | DOWN |
| chr | 4 | NONMMUG073888 | Linc | METAS | DOWN |
| chr | 4 | NONMMUG031407 | Linc | METAS | DOWN |
| chr | 4 | NONMMUG031454 | Exonic | METAS | DOWN |
| chr | 4 | NONMMUG031521 | Linc | METAS | DOWN |
| chr | 4 | NONMMUG031628 | Antisense | METAS | DOWN |
| chr | 4 | NONMMUG029161 | Sense no Exonic | METAS | UP |
| chr | 4 | NONMMUG029633 | Linc | METAS | UP |
| chr | 4 | NONMMUG029760 | Antisense | METAS | UP |
| chr | 4 | NONMMUG093421 | Antisense | METAS | UP |
| chr | 4 | NONMMUG030060 | Exonic | METAS | UP |
| chr | 4 | NONMMUG030377 | Linc | METAS | UP |
| chr | 4 | NONMMUG093198 | Linc | METAS | UP |
| chr | 4 | NONMMUG030660 | Linc | METAS | UP |
| chr | 4 | NONMMUG031059 | Linc | METAS | UP |
| chr | 4 | NONMMUG072689 | Linc | METAS | UP |
| chr | 4 | NONMMUG072727 | Linc | METAS | UP |
| chr | 4 | NONMMUG093280 | Linc | METAS | UP |
| chr | 4 | NONMMUG031539 | Exonic | METAS | UP |
| chr | 5 | NONMMUG034371 | Exonic | EMT | UP |
|  |  |  |  | MALIG | UP |
| chr | 5 | NONMMUG032788 | Sense no Exonic | MALIG | DOWN |
| chr | 5 | NONMMUG032975 | Exonic | MALIG | DOWN |
| chr | 5 | NONMMUG033312 | Antisense | MALIG | DOWN |
| chr | 5 | NONMMUG033549 | Antisense | MALIG | DOWN |
| chr | 5 | NONMMUG073966 | Linc | METAS | DOWN |
| chr | 5 | NONMMUG032250 | Linc | METAS | DOWN |
| chr | 5 | NONMMUG032747 | Linc | METAS | DOWN |
| chr | 5 | NONMMUG033280 | Sense no Exonic | METAS | DOWN |
| chr | 5 | NONMMUG033910 | Sense no Exonic | METAS | DOWN |
| chr | 5 | NONMMUG034306 | Linc | METAS | DOWN |
| chr | 5 | NONMMUG031822 | Linc | METAS | UP |
| chr | 5 | AI506816 | lncRNA | METAS | UP |
| chr | 5 | NONMMUG075356 | Linc | METAS | UP |
| chr | 5 | NONMMUG032375 | Exonic | METAS | UP |
| chr | 5 | NONMMUG075624 | Linc | METAS | UP |
| chr | 5 | NONMMUG032781 | Exonic | METAS | UP |
| chr | 5 | NONMMUG032929 | Exonic | METAS | UP |
| chr | 5 | NONMMUG033109 | Linc | METAS | UP |
| chr | 5 | NONMMUG033366 | Antisense | METAS | UP |
| chr | 5 | C330018A13Rik | lncRNA | METAS | UP |
| chr | 5 | NONMMUG074972 | Linc | METAS | UP |
| chr | 6 | NONMMUG034674 | Antisense | EMT | DOWN |
| chr | 6 | NONMMUG035585 | Linc | EMT | DOWN |
|  |  |  |  | MALIG | DOWN |
| chr | 6 | NONMMUG077727 | Antisense | EMT | DOWN |
| chr | 6 | NONMMUG035809 | Antisense | EMT | DOWN |
| chr | 6 | NONMMUG036055 | Linc | EMT | DOWN |
| chr | 6 | NONMMUG036111 | Sense no Exonic | EMT | DOWN |
| chr | 6 | NONMMUG036112 | Exonic | EMT | DOWN |
| chr | 6 | NONMMUG036478 | Exonic | EMT | DOWN |
| chr | 6 | NONMMUG036782 | Exonic | EMT | DOWN |
|  |  |  |  | METAS | UP |
| chr | 6 | NONMMUG035357 | Antisense | EMT | UP |
|  |  |  |  | MALIG | UP |
| chr | 6 | NONMMUG035347 | Exonic | MALIG | UP |
| chr | 6 | NONMMUG036638 | Exonic | MALIG | UP |
| chr | 6 | NONMMUG076345 | Linc | METAS | DOWN |
| chr | 6 | NONMMUG034969 | Exonic | METAS | DOWN |
| chr | 6 | NONMMUG034983 | Exonic | METAS | DOWN |
| chr | 6 | NONMMUG035144 | Linc | METAS | DOWN |
| chr | 6 | NONMMUG076838 | Linc | METAS | DOWN |
| chr | 6 | NONMMUG036011 | Linc | METAS | DOWN |
| chr | 6 | NONMMUG036610 | Sense no Exonic | METAS | DOWN |
| chr | 6 | NONMMUG094771 | Sense no Exonic | METAS | DOWN |
| chr | 6 | NONMMUG076323 | Linc | METAS | UP |
| chr | 6 | NONMMUG034529 | Sense no Exonic | METAS | UP |
| chr | 6 | NONMMUG034530 | Sense no Exonic | METAS | UP |
| chr | 6 | NONMMUG034532 | Linc | METAS | UP |
| chr | 6 | NONMMUG034537 | Linc | METAS | UP |
| chr | 6 | NONMMUG076374 | Linc | METAS | UP |
| chr | 6 | NONMMUG034772 | Sense no Exonic | METAS | UP |
| chr | 6 | 2210408F21Rik | lncRNA | METAS | UP |
| chr | 6 | NONMMUG035081 | Linc | METAS | UP |
| chr | 6 | NONMMUG035315 | Linc | METAS | UP |
| chr | 6 | NONMMUG036303 | Antisense | METAS | UP |
| chr | 6 | NONMMUG036584 | Linc | METAS | UP |
| chr | 6 | NONMMUG036585 | Linc | METAS | UP |
| chr | 6 | NONMMUG094443 | Linc | METAS | UP |
| chr | 7 | NONMMUG037484 | Linc | EMT | DOWN |
| chr | 7 | NONMMUG037656 | Linc | EMT | DOWN |
| chr | 7 | NONMMUG038825 | Exonic | MALIG | DOWN |
| chr | 7 | NONMMUG037169 | Linc | METAS | DOWN |
| chr | 7 | NONMMUG078422 | Linc | METAS | DOWN |
| chr | 7 | NONMMUG038280 | Sense no Exonic | METAS | DOWN |
| chr | 7 | NONMMUG039356 | Antisense | METAS | DOWN |
| chr | 7 | NONMMUG039685 | Antisense | METAS | DOWN |
| chr | 7 | NONMMUG078130 | Linc | METAS | UP |
| chr | 7 | NONMMUG037518 | Linc | METAS | UP |
| chr | 7 | NONMMUG037974 | Exonic | METAS | UP |
| chr | 7 | NONMMUG038093 | Linc | METAS | UP |
| chr | 7 | NONMMUG038156 | Linc | METAS | UP |
| chr | 7 | NONMMUG080056 | Linc | METAS | UP |
| chr | 7 | NONMMUG080057 | Linc | METAS | UP |
| chr | 7 | NONMMUG079196 | Linc | METAS | UP |
| chr | 7 | NONMMUG039413 | Sense no Exonic | METAS | UP |
| chr | 8 | NONMMUG040620 | Exonic | EMT | DOWN |
| chr | 8 | NONMMUG081006 | Linc | EMT | DOWN |
| chr | 8 | NONMMUG041885 | Linc | EMT | DOWN |
| chr | 8 | Gm42047 | lncRNA | EMT | UP |
| chr | 8 | NONMMUG040934 | Antisense | MALIG | DOWN |
| chr | 8 | NONMMUG081972 | Linc | METAS | DOWN |
| chr | 8 | NONMMUG040640 | Linc | METAS | DOWN |
| chr | 8 | Gm10033 | Processed transcript | METAS | DOWN |
| chr | 8 | Gm45899 | lncRNA | METAS | DOWN |
| chr | 8 | NONMMUG041516 | Exonic | METAS | DOWN |
| chr | 8 | NONMMUG041687 | Exonic | METAS | DOWN |
| chr | 8 | NONMMUG041761 | Linc | METAS | DOWN |
| chr | 8 | NONMMUG039970 | Linc | METAS | UP |
| chr | 8 | NONMMUG040211 | Linc | METAS | UP |
| chr | 8 | NONMMUG040267 | Exonic | METAS | UP |
| chr | 8 | NONMMUG095762 | Linc | METAS | UP |
| chr | 8 | NONMMUG095519 | Linc | METAS | UP |
| chr | 8 | NONMMUG040711 | Linc | METAS | UP |
| chr | 8 | NONMMUG041144 | Exonic | METAS | UP |
| chr | 8 | NONMMUG081486 | Linc | METAS | UP |
| chr | 8 | NONMMUG095675 | Linc | METAS | UP |
| chr | 9 | NONMMUG043682 | Antisense | EMT | DOWN |
| chr | 9 | NONMMUG043940 | Antisense | EMT | DOWN |
| chr | 9 | 4930461G14Rik | lncRNA | EMT | UP |
| chr | 9 | NONMMUG043691 | Linc | EMT | UP |
|  |  |  |  | MALIG | UP |
| chr | 9 | NONMMUG084653 | Linc | MALIG | DOWN |
| chr | 9 | Gm9531 | Processed transcript | MALIG | UP |
| chr | 9 | NONMMUG042010 | Linc | METAS | DOWN |
| chr | 9 | NONMMUG083078 | Linc | METAS | DOWN |
| chr | 9 | Mir100hg | lncRNA | METAS | DOWN |
| chr | 9 | NONMMUG042550 | Linc | METAS | DOWN |
| chr | 9 | NONMMUG042548 | Linc | METAS | DOWN |
| chr | 9 | NONMMUG042574 | Linc | METAS | DOWN |
| chr | 9 | NONMMUG084600 | Linc | METAS | DOWN |
| chr | 9 | NONMMUG042613 | Linc | METAS | DOWN |
| chr | 9 | NONMMUG042614 | Linc | METAS | DOWN |
| chr | 9 | NONMMUG042674 | Sense no Exonic | METAS | DOWN |
| chr | 9 | NONMMUG042681 | Sense no Exonic | METAS | DOWN |
| chr | 9 | NONMMUG042714 | Linc | METAS | DOWN |
| chr | 9 | NONMMUG043117 | Linc | METAS | DOWN |
| chr | 9 | NONMMUG084882 | Linc | METAS | DOWN |
| chr | 9 | NONMMUG042232 | Exonic | METAS | UP |
| chr | 9 | NONMMUG084497 | Linc | METAS | UP |
| chr | 9 | NONMMUG084499 | Linc | METAS | UP |
| chr | 9 | NONMMUG042534 | Exonic | METAS | UP |
| chr | 9 | NONMMUG096085 | Linc | METAS | UP |
| chr | 9 | NONMMUG043191 | Sense no Exonic | METAS | UP |
| chr | 9 | NONMMUG043328 | Linc | METAS | UP |
| chr | 9 | NONMMUG085249 | Linc | METAS | UP |
| chr | 9 | NONMMUG084029 | Linc | METAS | UP |
| chr | 9 | NONMMUG044238 | Linc | METAS | UP |
| chr | 9 | NONMMUG044343 | Linc | METAS | UP |
| chr | 10 | NONMMUG050805 | Linc | METAS | DOWN |
| chr | 10 | NONMMUG003396 | Linc | METAS | DOWN |
| chr | 10 | NONMMUG051177 | Linc | METAS | DOWN |
| chr | 10 | NONMMUG051178 | Linc | METAS | DOWN |
| chr | 10 | NONMMUG004760 | Linc | METAS | DOWN |
| chr | 10 | NONMMUG003191 | Linc | METAS | UP |
| chr | 10 | NONMMUG003196 | Linc | METAS | UP |
| chr | 10 | NONMMUG003214 | Linc | METAS | UP |
| chr | 10 | NONMMUG003654 | Linc | METAS | UP |
| chr | 10 | NONMMUG003708 | Linc | METAS | UP |
| chr | 10 | NONMMUG003763 | Linc | METAS | UP |
| chr | 10 | NONMMUG051210 | Linc | METAS | UP |
| chr | 10 | NONMMUG004180 | Linc | METAS | UP |
| chr | 10 | NONMMUG004241 | Linc | METAS | UP |
| chr | 10 | NONMMUG051350 | Linc | METAS | UP |
| chr | 10 | NONMMUG051351 | Linc | METAS | UP |
| chr | 10 | NONMMUG004995 | Linc | METAS | UP |
| chr | 11 | NONMMUG005701 | Linc | EMT | UP |
| chr | 11 | NONMMUG005765 | Linc | EMT | UP |
| chr | 11 | NONMMUG006923 | Linc | EMT | UP |
| chr | 11 | NONMMUG007059 | Linc | EMT | UP |
| chr | 11 | NONMMUG007358 | Linc | EMT | UP |
| chr | 11 | NONMMUG007404 | Linc | EMT | UP |
| chr | 11 | NONMMUG007405 | Linc | EMT | UP |
| chr | 11 | NONMMUG007415 | Linc | EMT | UP |
| chr | 11 | NONMMUG007610 | Linc | EMT | UP |
| chr | 11 | NONMMUG006067 | Linc | METAS | DOWN |
| chr | 11 | Olfr1372-ps1 | Processed transcript | METAS | DOWN |
| chr | 11 | NONMMUG007011 | Linc | METAS | DOWN |
| chr | 11 | NONMMUG007141 | Linc | METAS | DOWN |
| chr | 11 | NONMMUG007448 | Linc | METAS | DOWN |
| chr | 11 | NONMMUG005219 | Linc | METAS | UP |
| chr | 11 | Snhg15 | lncRNA | METAS | UP |
| chr | 11 | NONMMUG005345 | Linc | METAS | UP |
| chr | 11 | NONMMUG005721 | Linc | METAS | UP |
| chr | 11 | 4933415A04Rik | lncRNA | METAS | UP |
| chr | 11 | NONMMUG005976 | Linc | METAS | UP |
| chr | 11 | NONMMUG006050 | Linc | METAS | UP |
| chr | 11 | NONMMUG052084 | Linc | METAS | UP |
| chr | 11 | NONMMUG006346 | Linc | METAS | UP |
| chr | 11 | NONMMUG053148 | Linc | METAS | UP |
| chr | 11 | Gm11427 | Processed transcript | METAS | UP |
| chr | 11 | NONMMUG007014 | Linc | METAS | UP |
| chr | 11 | NONMMUG007171 | Linc | METAS | UP |
| chr | 11 | NONMMUG007554 | Linc | METAS | UP |
| chr | 11 | NONMMUG052545 | Linc | METAS | UP |
| chr | 11 | NONMMUG089034 | Linc | METAS | UP |
| chr | 11 | NONMMUG007819 | Linc | METAS | UP |
| chr | 11 | NONMMUG007952 | Linc | METAS | UP |
| chr | 11 | NONMMUG008067 | Linc | METAS | UP |
| chr | 11 | NONMMUG089078 | Linc | METAS | UP |
| chr | 12 | NONMMUG008775 | Linc | MALIG | DOWN |
| chr | 12 | NONMMUG055082 | Linc | METAS | DOWN |
| chr | 12 | NONMMUG055083 | Linc | METAS | DOWN |
| chr | 12 | NONMMUG055084 | Linc | METAS | DOWN |
| chr | 12 | NONMMUG055192 | Linc | METAS | DOWN |
| chr | 12 | NONMMUG055193 | Linc | METAS | DOWN |
| chr | 12 | Gm49327 | Processed transcript | METAS | UP |
| chr | 12 | NONMMUG053817 | Linc | METAS | UP |
| chr | 12 | 1700030C10Rik | Processed transcript | METAS | UP |
| chr | 12 | 3110053B16Rik | Processed transcript | METAS | UP |
| chr | 12 | NONMMUG089378 | Linc | METAS | UP |
| chr | 12 | NONMMUG008678 | Linc | METAS | UP |
| chr | 12 | NONMMUG089188 | Linc | METAS | UP |
| chr | 12 | NONMMUG009325 | Linc | METAS | UP |
| chr | 12 | NONMMUG054375 | Linc | METAS | UP |
| chr | 12 | NONMMUG009847 | Linc | METAS | UP |
| chr | 12 | NONMMUG009943 | Linc | METAS | UP |
| chr | 12 | Gm30948 | lncRNA | METAS | UP |
| chr | 13 | Gpr137b-ps | Processed transcript | EMT | DOWN |
|  |  |  |  | METAS | UP |
| chr | 13 | NONMMUG010281 | Linc | EMT | UP |
| chr | 13 | Gm48682 | lncRNA | EMT | UP |
| chr | 13 | NONMMUG010899 | Linc | EMT | UP |
| chr | 13 | NONMMUG011236 | Linc | EMT | UP |
| chr | 13 | Gm47486 | lncRNA | METAS | DOWN |
| chr | 13 | Gm48754 | lncRNA | METAS | DOWN |
| chr | 13 | Gm48799 | lncRNA | METAS | DOWN |
| chr | 13 | NONMMUG010423 | Linc | METAS | DOWN |
| chr | 13 | NONMMUG056722 | Linc | METAS | DOWN |
| chr | 13 | NONMMUG056723 | Linc | METAS | DOWN |
| chr | 13 | NONMMUG056949 | Linc | METAS | DOWN |
| chr | 13 | NONMMUG010169 | Linc | METAS | UP |
| chr | 13 | 2810429I04Rik | lncRNA | METAS | UP |
| chr | 13 | NONMMUG010211 | Linc | METAS | UP |
| chr | 13 | NONMMUG010555 | Linc | METAS | UP |
| chr | 13 | NONMMUG089618 | Linc | METAS | UP |
| chr | 13 | NONMMUG089844 | Linc | METAS | UP |
| chr | 13 | NONMMUG011033 | Linc | METAS | UP |
| chr | 13 | NONMMUG011252 | Linc | METAS | UP |
| chr | 13 | NONMMUG011344 | Linc | METAS | UP |
| chr | 13 | 3110070M22Rik | lncRNA | METAS | UP |
| chr | 14 | NONMMUG012806 | Linc | EMT | UP |
| chr | 14 | Gm43305 | lncRNA | EMT | UP |
| chr | 14 | NONMMUG013015 | Linc | EMT | UP |
| chr | 14 | Gm20687 | lncRNA | EMT | UP |
| chr | 14 | NONMMUG012182 | Linc | MALIG | DOWN |
| chr | 14 | NONMMUG057571 | Linc | MALIG | DOWN |
| chr | 14 | 9330188P03Rik | lncRNA | MALIG | DOWN |
| chr | 14 | NONMMUG012176 | Linc | METAS | DOWN |
| chr | 14 | NONMMUG012433 | Linc | METAS | DOWN |
| chr | 14 | NONMMUG012443 | Linc | METAS | DOWN |
| chr | 14 | NONMMUG012555 | Linc | METAS | DOWN |
| chr | 14 | NONMMUG012931 | Linc | METAS | DOWN |
| chr | 14 | NONMMUG013142 | Linc | METAS | DOWN |
| chr | 14 | NONMMUG090316 | Linc | METAS | DOWN |
| chr | 14 | NONMMUG013173 | Linc | METAS | DOWN |
| chr | 14 | NONMMUG013305 | Linc | METAS | DOWN |
| chr | 14 | NONMMUG013390 | Linc | METAS | DOWN |
| chr | 14 | Gm10110 | Processed transcript | METAS | DOWN |
| chr | 14 | NONMMUG013880 | Linc | METAS | DOWN |
| chr | 14 | Gm31517 | lncRNA | METAS | UP |
| chr | 14 | NONMMUG012801 | Linc | METAS | UP |
| chr | 14 | NONMMUG013259 | Linc | METAS | UP |
| chr | 14 | NONMMUG013286 | Linc | METAS | UP |
| chr | 14 | NONMMUG013551 | Linc | METAS | UP |
| chr | 14 | NONMMUG059207 | Linc | METAS | UP |
| chr | 14 | NONMMUG013808 | Linc | METAS | UP |
| chr | 15 | NONMMUG060759 | Linc | EMT | DOWN |
| chr | 15 | NONMMUG060945 | Linc | EMT | DOWN |
| chr | 15 | NONMMUG060946 | Linc | EMT | DOWN |
| chr | 15 | NONMMUG015514 | Linc | EMT | DOWN |
|  |  |  |  | METAS | UP |
| chr | 15 | Gm48957 | lncRNA | EMT | UP |
| chr | 15 | NONMMUG014171 | Linc | EMT | UP |
| chr | 15 | NONMMUG014649 | Linc | EMT | UP |
| chr | 15 | NONMMUG014882 | Linc | EMT | UP |
| chr | 15 | NONMMUG015352 | Linc | EMT | UP |
| chr | 15 | NONMMUG015380 | Linc | EMT | UP |
| chr | 15 | NONMMUG015565 | Linc | EMT | UP |
| chr | 15 | NONMMUG014060 | Linc | METAS | DOWN |
| chr | 15 | NONMMUG014061 | Linc | METAS | DOWN |
| chr | 15 | NONMMUG014496 | Linc | METAS | DOWN |
| chr | 15 | NONMMUG014497 | Linc | METAS | DOWN |
| chr | 15 | NONMMUG014501 | Linc | METAS | DOWN |
| chr | 15 | NONMMUG014605 | Linc | METAS | DOWN |
| chr | 15 | NONMMUG014607 | Linc | METAS | DOWN |
| chr | 15 | NONMMUG014613 | Linc | METAS | DOWN |
| chr | 15 | NONMMUG059968 | Linc | METAS | DOWN |
| chr | 15 | NONMMUG014784 | Linc | METAS | DOWN |
| chr | 15 | NONMMUG014888 | Linc | METAS | DOWN |
| chr | 15 | NONMMUG014959 | Linc | METAS | DOWN |
| chr | 15 | NONMMUG014968 | Linc | METAS | DOWN |
| chr | 15 | NONMMUG059440 | Linc | METAS | UP |
| chr | 15 | NONMMUG014167 | Linc | METAS | UP |
| chr | 15 | NONMMUG059735 | Linc | METAS | UP |
| chr | 15 | NONMMUG059955 | Linc | METAS | UP |
| chr | 15 | Gm49497 | lncRNA | METAS | UP |
| chr | 15 | NONMMUG014636 | Linc | METAS | UP |
| chr | 15 | NONMMUG014744 | Linc | METAS | UP |
| chr | 15 | NONMMUG060096 | Linc | METAS | UP |
| chr | 15 | NONMMUG015364 | Linc | METAS | UP |
| chr | 15 | NONMMUG061049 | Linc | METAS | UP |
| chr | 15 | NONMMUG015409 | Linc | METAS | UP |
| chr | 16 | Mx1 | Processed transcript | EMT | DOWN |
|  |  |  |  | MALIG | DOWN |
| chr | 16 | NONMMUG016086 | Linc | EMT | UP |
| chr | 16 | NONMMUG016679 | Linc | EMT | UP |
| chr | 16 | NONMMUG017258 | Linc | EMT | UP |
| chr | 16 | NONMMUG016694 | Linc | MALIG | DOWN |
| chr | 16 | NONMMUG016698 | Linc | MALIG | DOWN |
| chr | 16 | NONMMUG016700 | Linc | MALIG | DOWN |
| chr | 16 | NONMMUG016702 | Linc | MALIG | DOWN |
| chr | 16 | NONMMUG017293 | Linc | MALIG | DOWN |
| chr | 16 | NONMMUG016182 | Linc | METAS | DOWN |
| chr | 16 | NONMMUG016322 | Linc | METAS | DOWN |
| chr | 16 | NONMMUG016861 | Linc | METAS | DOWN |
| chr | 16 | NONMMUG017042 | Linc | METAS | DOWN |
| chr | 16 | NONMMUG017150 | Linc | METAS | DOWN |
| chr | 16 | NONMMUG017180 | Linc | METAS | DOWN |
| chr | 16 | NONMMUG017182 | Linc | METAS | DOWN |
| chr | 16 | NONMMUG016352 | Linc | METAS | UP |
| chr | 16 | NONMMUG016391 | Linc | METAS | UP |
| chr | 16 | Gm49701 | lncRNA | METAS | UP |
| chr | 16 | NONMMUG016826 | Linc | METAS | UP |
| chr | 16 | NONMMUG017009 | Linc | METAS | UP |
| chr | 17 | NONMMUG091337 | Linc | EMT | DOWN |
| chr | 17 | NONMMUG017999 | Linc | EMT | UP |
| chr | 17 | NONMMUG018123 | Linc | EMT | UP |
| chr | 17 | NONMMUG018213 | Linc | EMT | UP |
| chr | 17 | NONMMUG019046 | Linc | EMT | UP |
| chr | 17 | NONMMUG019129 | Linc | EMT | UP |
| chr | 17 | NONMMUG017903 | Linc | MALIG | DOWN |
| chr | 17 | NONMMUG017389 | Linc | METAS | DOWN |
| chr | 17 | NONMMUG017413 | Linc | METAS | DOWN |
| chr | 17 | NONMMUG063023 | Linc | METAS | DOWN |
| chr | 17 | NONMMUG017678 | Linc | METAS | DOWN |
| chr | 17 | NONMMUG017995 | Linc | METAS | DOWN |
| chr | 17 | NONMMUG018156 | Linc | METAS | DOWN |
| chr | 17 | NONMMUG018812 | Linc | METAS | DOWN |
| chr | 17 | NONMMUG018814 | Linc | METAS | DOWN |
| chr | 17 | NONMMUG091316 | Linc | METAS | DOWN |
| chr | 17 | NONMMUG018344 | Linc | METAS | UP |
| chr | 17 | NONMMUG064070 | Linc | METAS | UP |
| chr | 17 | NONMMUG018843 | Linc | METAS | UP |
| chr | 17 | Trmt61b | lncRNA | METAS | UP |
| chr | 18 | NONMMUG019417 | Linc | EMT | DOWN |
|  |  |  |  | MALIG | DOWN |
| chr | 18 | NONMMUG020007 | Linc | EMT | UP |
| chr | 18 | NONMMUG020083 | Linc | EMT | UP |
| chr | 18 | NONMMUG020347 | Linc | EMT | UP |
| chr | 18 | NONMMUG019357 | Linc | MALIG | DOWN |
| chr | 18 | NONMMUG019647 | Linc | MALIG | DOWN |
| chr | 18 | NONMMUG019483 | Linc | METAS | DOWN |
| chr | 18 | NONMMUG019487 | Linc | METAS | DOWN |
| chr | 18 | Zscan30 | Processed transcript | METAS | DOWN |
| chr | 18 | NONMMUG019566 | Linc | METAS | DOWN |
| chr | 18 | NONMMUG019722 | Linc | METAS | DOWN |
| chr | 18 | NONMMUG064790 | Linc | METAS | DOWN |
| chr | 18 | NONMMUG019543 | Linc | METAS | UP |
| chr | 18 | NONMMUG064786 | Linc | METAS | UP |
| chr | 18 | Gm41760 | lncRNA | METAS | UP |
| chr | 18 | AC132307.1 | lncRNA | METAS | UP |
| chr | 19 | NONMMUG021256 | Linc | EMT | UP |
| chr | 19 | NONMMUG021327 | Linc | EMT | UP |
| chr | 19 | NONMMUG021331 | Linc | EMT | UP |
| chr | 19 | NONMMUG021544 | Linc | EMT | UP |
| chr | 19 | NONMMUG021602 | Linc | EMT | UP |
| chr | 19 | NONMMUG021688 | Linc | EMT | UP |
| chr | 19 | NONMMUG020553 | Linc | METAS | DOWN |
| chr | 19 | NONMMUG020586 | Linc | METAS | DOWN |
| chr | 19 | NONMMUG020673 | Linc | METAS | DOWN |
| chr | 19 | NONMMUG065760 | Linc | METAS | DOWN |
| chr | 19 | NONMMUG020855 | Linc | METAS | DOWN |
| chr | 19 | Gm50216 | lncRNA | METAS | DOWN |
| chr | 19 | NONMMUG066351 | Linc | METAS | DOWN |
| chr | 19 | NONMMUG065931 | Linc | METAS | DOWN |
| chr | 19 | NONMMUG092026 | Linc | METAS | DOWN |
| chr | 19 | NONMMUG091924 | Linc | METAS | UP |
| chr | 19 | NONMMUG020717 | Linc | METAS | UP |
| chr | 19 | Gm14964 | lncRNA | METAS | UP |
| chr | 19 | NONMMUG021044 | Linc | METAS | UP |
| chr | 19 | NONMMUG021229 | Linc | METAS | UP |
| chr | 19 | NONMMUG021352 | Linc | METAS | UP |
| chr | 19 | NONMMUG091984 | Linc | METAS | UP |
| chr | X | NONMMUG044486 | Linc | EMT | DOWN |
| chr | X | NONMMUG044909 | Linc | EMT | DOWN |
| chr | X | Xist | lncRNA | MALIG | DOWN |
| chr | X | NONMMUG045569 | Linc | MALIG | DOWN |
| chr | X | NONMMUG044425 | Linc | METAS | DOWN |
| chr | X | NONMMUG044889 | Linc | METAS | DOWN |
| chr | X | NONMMUG096512 | Linc | METAS | DOWN |
| chr | X | NONMMUG045574 | Linc | METAS | UP |
| chr | X | NONMMUG045946 | Linc | METAS | UP |
| chr | X | NONMMUG045988 | Linc | METAS | UP |
| chr | X | NONMMUG046218 | Linc | METAS | UP |
| chr | X | NONMMUG046220 | Linc | METAS | UP |
| chr | Y | NONMMUG096601 | Linc | METAS | DOWN |
| chr | Y | Gm47283 | lncRNA | METAS | DOWN |
| chr | Y | NONMMUG046461 | Linc | METAS | UP |
