# Supplementary figures and images for: LncRNA Dlx4os drives malignant transformation and phenotype switching in melanoma
Source: Epigenetics. 2026 Mar 19;21(1):2641924. doi: 10.1080/15592294.2026.2641924 (PMC13003849; doi:10.1080/15592294.2026.2641924)

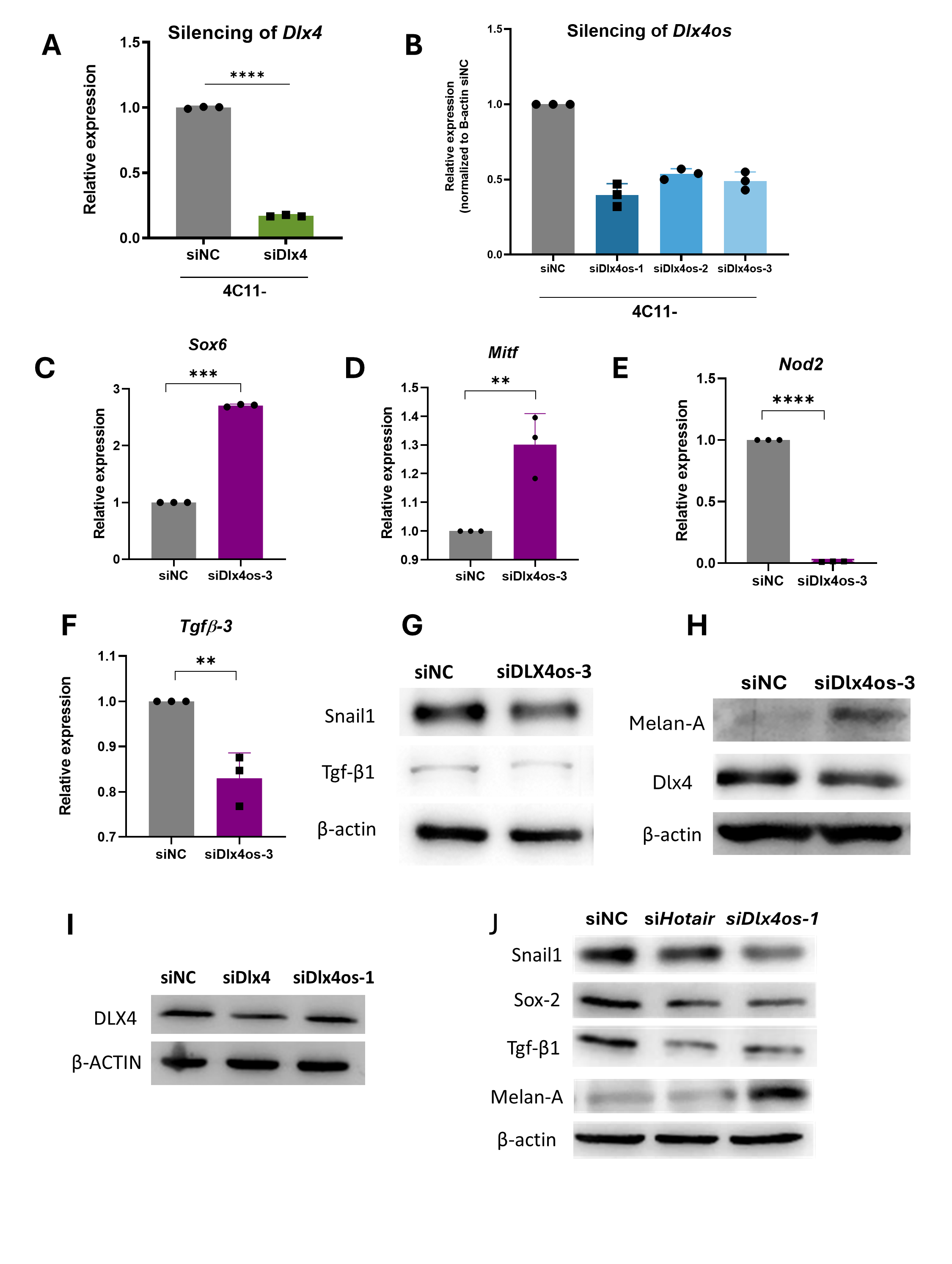

Supplement: Figure S1.TIF [file KEPI_A_2641924_SM4555.tif]

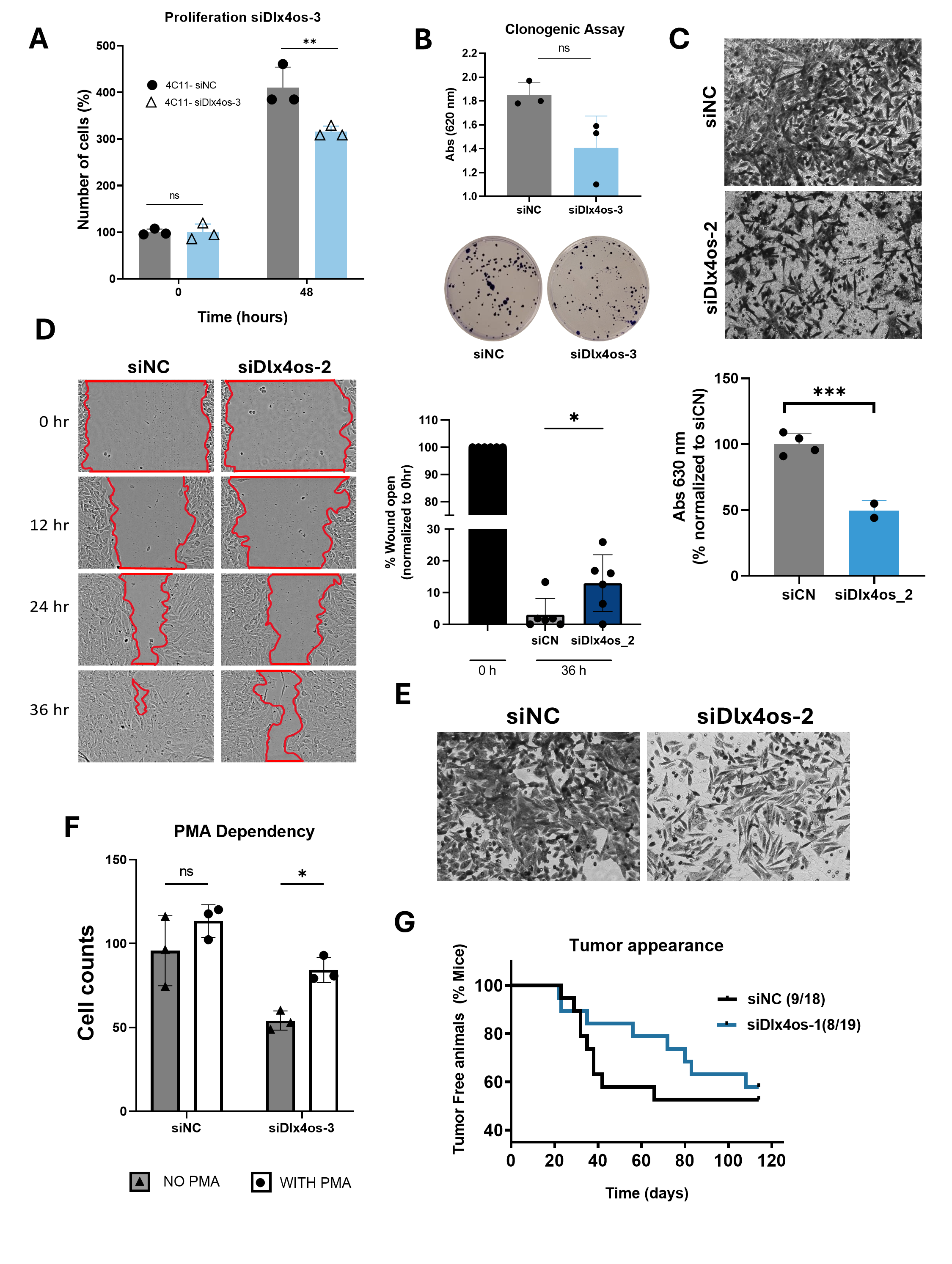

Supplement: Figure S2.TIF [file KEPI_A_2641924_SM4554.tif]
