# Supplementary material for: LncRNA Dlx4os drives malignant transformation and phenotype switching in melanoma
Source: Epigenetics. 2026 Mar 19;21(1):2641924. doi: 10.1080/15592294.2026.2641924 (PMC13003849; doi:10.1080/15592294.2026.2641924)
Supplement: Supplementary Material.docx [file KEPI_A_2641924_SM4553.docx]

**Supplementary Material**

**Table 1. Primers used for RT-qPCR**

| **Primer** | **Specie** | **Foward** | **Reverse** |
| --- | --- | --- | --- |
| Beta-actina | mouse | ACCGTGAAAAGATGACCAG | GTACGCCAGACGCATACAG |
| Dlx4OS | mouse | CTAATCCTGCCTCCACCCTG | CTCCATGAGGGTCTGTTGGT |
| Mitf | mouse | CCTATGGCTATGCTCACTCTT | GTTCATACCTGGGCACTCAC |
| Nod2 | mouse | CCTAGCACTGATGCTGGAGAAG | CGGTAGGTGATGCCATTGTTGG |
| Sox10 | mouse | TCAGAGTTAGCATGGCACGG | AAGGGTGCAAGGCAAAGGTA |
| Sox6 | mouse | GCATAAGTGACCGTTTTGGCAGG | GGCATCTTTGCTCCAGGTGACA |
| Tgfb3 | mouse | AAGCAGCGCTACATAGGTGGCA | GGCTGAAAGGTGTGACATGGAC |
| U6 | mouse | AGGGCTACCCAGTGTTCTGA | TGTGGAACACTACATGAATTTGC |
| Rpl19 | mouse | GAAATCGCCAATGCCAACTC | CTTCCCTATGCCCATATG CC |
| mlana | mouse | GACGAAGTGGATACAGAACCTTG | CTCTTGAGAAGACAGTCGGCTG |
| GAPDH | human | ACCTGACCTGCCGTCTAGAA | GTCAAAGGTGGAGGAGTGGG |
| HSALNT | human | AGGGCCATGAAACTTTGAGGAGC | ACAAGCCCTTAACCAGCCCAA |

**Table 2. Antibodies used for western blotting**

| **Primary** | | |  | **Secundary** | |
| --- | --- | --- | --- | --- | --- |
| **Antibody** | **Catalog** | **Molecular weigh** |  | **Antibody** | **Catalog** |
| vinculin | Cell Signaling #4650 | 128 kDa |  | anti-rabbit | KPL #04-15-06 |
| lamin A/C | Santa Cruz #6215 | 68-72 kDa |  | anti-goat | KPL #14-13-06 |
| Snail | Cell Signalling #3879 | 29kDa |  | anti-rabbit | KPL #04-15-06 |
| Sox2 | Abgent AM2048A | 38kDa |  | anti-mouse | Biorad #1706516 |
| TgB1 | Santa Cruz #130348 | 25/14 kDa |  | anti-rabbit | KPL #04-15-06 |
| mlana | Santa Cruz #20032 | 23kDa |  | anti-mouse | Biorad #1706516 |
| B-actina | Abgene AM1829b | 42 kDa |  | anti-mouse | Biorad #1706516 |

**Table 3. lncRNAs filtered by signatures (501)**

| chr | | TRANSCRIPT NAME | LncRNA Class | SIGNATURE | DIRECTION |
| --- | --- | --- | --- | --- | --- |
| chr | 1 | NONMMUG048626 | Linc | EMT | DOWN |
| chr | 1 | NONMMUG047617 | Linc | EMT | DOWN |
| chr | 1 | NONMMUG000567 | Linc | EMT | UP |
| chr | 1 | NONMMUG000991 | Exonic | EMT | UP |
| chr | 1 | Gm29100 | lncRNA | EMT | UP |
| chr | 1 | NONMMUG001361 | Sense no Exonic | EMT | UP |
| chr | 1 | NONMMUG001822 | Linc | EMT | UP |
| chr | 1 | NONMMUG002548 | Linc | MALIG | DOWN |
| chr | 1 | NONMMUG047671 | Linc | MALIG | DOWN |
| chr | 1 | NONMMUG046632 | Sense no Exonic | METAS | DOWN |
| chr | 1 | NONMMUG047121 | Sense no Exonic | METAS | DOWN |
| chr | 1 | NONMMUG047199 | Linc | METAS | DOWN |
| chr | 1 | NONMMUG087678 | Linc | METAS | DOWN |
| chr | 1 | NONMMUG001887 | Linc | METAS | DOWN |
| chr | 1 | NONMMUG002471 | Linc | METAS | DOWN |
| chr | 1 | NONMMUG049435 | Linc | METAS | DOWN |
| chr | 1 | NONMMUG087584 | Linc | METAS | UP |
| chr | 1 | NONMMUG000420 | Exonic | METAS | UP |
| chr | 1 | NONMMUG048348 | Antisense | METAS | UP |
| chr | 1 | NONMMUG046996 | Linc | METAS | UP |
| chr | 1 | NONMMUG001327 | Antisense | METAS | UP |
| chr | 1 | NONMMUG001466 | Linc | METAS | UP |
| chr | 1 | NONMMUG001638 | Linc | METAS | UP |
| chr | 1 | NONMMUG001660 | Linc | METAS | UP |
| chr | 1 | NONMMUG047457 | Linc | METAS | UP |
| chr | 1 | NONMMUG047536 | Antisense | METAS | UP |
| chr | 1 | NONMMUG002594 | Exonic | METAS | UP |
| chr | 2 | NONMMUG066689 | Linc | EMT | DOWN |
| chr | 2 | NONMMUG092122 | Exonic | EMT | DOWN |
| chr | 2 | NONMMUG024494 | Sense no Exonic | EMT | DOWN |
| chr | 2 | NONMMUG068874 | Linc | EMT | DOWN |
| chr | 2 | NONMMUG025550 | Antisense | EMT | DOWN |
| chr | 2 | NONMMUG025564 | Antisense | EMT | DOWN |
| chr | 2 | NONMMUG022340 | Linc | EMT | UP |
| chr | 2 | NONMMUG022562 | Linc | EMT | UP |
| chr | 2 | NONMMUG022684 | Linc | EMT | UP |
| chr | 2 | NONMMUG023124 | Antisense | EMT | UP |
| chr | 2 | NONMMUG023721 | Sense no Exonic | EMT | UP |
| chr | 2 | NONMMUG023848 | Antisense | EMT | UP |
| chr | 2 | NONMMUG025670 | Linc | MALIG | UP |
| chr | 2 | NONMMUG021809 | Sense no Exonic | METAS | DOWN |
| chr | 2 | NONMMUG066597 | Antisense | METAS | DOWN |
| chr | 2 | NONMMUG022390 | Linc | METAS | DOWN |
| chr | 2 | NONMMUG022413 | Antisense | METAS | DOWN |
| chr | 2 | NONMMUG022518 | Linc | METAS | DOWN |
| chr | 2 | NONMMUG023099 | Exonic | METAS | DOWN |
| chr | 2 | NONMMUG023476 | Linc | METAS | DOWN |
| chr | 2 | NONMMUG023935 | Linc | METAS | DOWN |
| chr | 2 | NONMMUG024175 | Sense no Exonic | METAS | DOWN |
| chr | 2 | NONMMUG024302 | Linc | METAS | DOWN |
| chr | 2 | NONMMUG024452 | Exonic | METAS | DOWN |
| chr | 2 | NONMMUG024479 | Linc | METAS | DOWN |
| chr | 2 | NONMMUG024539 | Exonic | METAS | DOWN |
| chr | 2 | NONMMUG024578 | Sense no Exonic | METAS | DOWN |
| chr | 2 | NONMMUG025090 | Linc | METAS | DOWN |
| chr | 2 | NONMMUG025486 | Exonic | METAS | DOWN |
| chr | 2 | NONMMUG025574 | Linc | METAS | DOWN |
| chr | 2 | NONMMUG068887 | Linc | METAS | DOWN |
| chr | 2 | NONMMUG025748 | Linc | METAS | DOWN |
| chr | 2 | NONMMUG092620 | Linc | METAS | DOWN |
| chr | 2 | NONMMUG025749 | Linc | METAS | DOWN |
| chr | 2 | NONMMUG025750 | Antisense | METAS | DOWN |
| chr | 2 | NONMMUG025980 | Linc | METAS | DOWN |
| chr | 2 | NONMMUG022181 | Linc | METAS | UP |
| chr | 2 | NONMMUG022255 | Exonic | METAS | UP |
| chr | 2 | NONMMUG022268 | Antisense | METAS | UP |
| chr | 2 | NONMMUG022454 | Exonic | METAS | UP |
| chr | 2 | NONMMUG023068 | Sense no Exonic | METAS | UP |
| chr | 2 | NONMMUG023451 | Antisense | METAS | UP |
| chr | 2 | NONMMUG023453 | Sense no Exonic | METAS | UP |
| chr | 2 | NONMMUG023561 | Exonic | METAS | UP |
| chr | 2 | NONMMUG023593 | Linc | METAS | UP |
| chr | 2 | NONMMUG067176 | Linc | METAS | UP |
| chr | 2 | NONMMUG023633 | Linc | METAS | UP |
| chr | 2 | NONMMUG023937 | Linc | METAS | UP |
| chr | 2 | NONMMUG024232 | Sense no Exonic | METAS | UP |
| chr | 2 | NONMMUG024278 | Linc | METAS | UP |
| chr | 2 | NONMMUG024510 | Exonic | METAS | UP |
| chr | 2 | NONMMUG024511 | Exonic | METAS | UP |
| chr | 2 | NONMMUG024712 | Antisense | METAS | UP |
| chr | 2 | NONMMUG025066 | Linc | METAS | UP |
| chr | 2 | NONMMUG025251 | Sense no Exonic | METAS | UP |
| chr | 2 | NONMMUG025546 | Linc | METAS | UP |
| chr | 2 | NONMMUG025703 | Linc | METAS | UP |
| chr | 3 | NONMMUG027430 | Exonic | EMT | DOWN |
| chr | 3 | NONMMUG070920 | Antisense | METAS | DOWN |
| chr | 3 | NONMMUG026453 | Linc | METAS | DOWN |
| chr | 3 | NONMMUG027079 | Exonic | METAS | DOWN |
| chr | 3 | NONMMUG071245 | Linc | METAS | DOWN |
| chr | 3 | NONMMUG027443 | Linc | METAS | DOWN |
| chr | 3 | NONMMUG028241 | Linc | METAS | DOWN |
| chr | 3 | NONMMUG026551 | Linc | METAS | UP |
| chr | 3 | NONMMUG026552 | Linc | METAS | UP |
| chr | 3 | NONMMUG027012 | Antisense | METAS | UP |
| chr | 3 | NONMMUG027093 | Linc | METAS | UP |
| chr | 3 | Gm15417 | lncRNA | METAS | UP |
| chr | 3 | NONMMUG027312 | Antisense | METAS | UP |
| chr | 3 | NONMMUG092958 | Linc | METAS | UP |
| chr | 3 | NONMMUG071247 | Linc | METAS | UP |
| chr | 3 | Gm5546 | lncRNA | METAS | UP |
| chr | 3 | NONMMUG070111 | Sense no Exonic | METAS | UP |
| chr | 4 | NONMMUG028483 | Exonic | EMT | DOWN |
|  |  |  |  | METAS | UP |
| chr | 4 | NONMMUG028975 | Antisense | EMT | DOWN |
| chr | 4 | NONMMUG029165 | Exonic | EMT | DOWN |
| chr | 4 | NONMMUG072477 | Linc | EMT | DOWN |
| chr | 4 | NONMMUG029064 | Linc | MALIG | DOWN |
| chr | 4 | NONMMUG029562 | Linc | MALIG | DOWN |
| chr | 4 | Gm49890 | lncRNA | MALIG | DOWN |
| chr | 4 | NONMMUG029945 | Linc | MALIG | DOWN |
| chr | 4 | NONMMUG029954 | Sense no Exonic | MALIG | DOWN |
| chr | 4 | NONMMUG029955 | Antisense | MALIG | DOWN |
| chr | 4 | NONMMUG072825 | Linc | METAS | DOWN |
| chr | 4 | NONMMUG028686 | Linc | METAS | DOWN |
| chr | 4 | NONMMUG028863 | Exonic | METAS | DOWN |
| chr | 4 | NONMMUG029073 | Linc | METAS | DOWN |
| chr | 4 | NONMMUG093375 | Linc | METAS | DOWN |
| chr | 4 | NONMMUG029813 | Antisense | METAS | DOWN |
| chr | 4 | NONMMUG029868 | Antisense | METAS | DOWN |
| chr | 4 | NONMMUG031169 | Linc | METAS | DOWN |
| chr | 4 | NONMMUG072694 | Linc | METAS | DOWN |
| chr | 4 | NONMMUG031291 | Linc | METAS | DOWN |
| chr | 4 | NONMMUG073887 | Linc | METAS | DOWN |
| chr | 4 | NONMMUG073888 | Linc | METAS | DOWN |
| chr | 4 | NONMMUG031407 | Linc | METAS | DOWN |
| chr | 4 | NONMMUG031454 | Exonic | METAS | DOWN |
| chr | 4 | NONMMUG031521 | Linc | METAS | DOWN |
| chr | 4 | NONMMUG031628 | Antisense | METAS | DOWN |
| chr | 4 | NONMMUG029161 | Sense no Exonic | METAS | UP |
| chr | 4 | NONMMUG029633 | Linc | METAS | UP |
| chr | 4 | NONMMUG029760 | Antisense | METAS | UP |
| chr | 4 | NONMMUG093421 | Antisense | METAS | UP |
| chr | 4 | NONMMUG030060 | Exonic | METAS | UP |
| chr | 4 | NONMMUG030377 | Linc | METAS | UP |
| chr | 4 | NONMMUG093198 | Linc | METAS | UP |
| chr | 4 | NONMMUG030660 | Linc | METAS | UP |
| chr | 4 | NONMMUG031059 | Linc | METAS | UP |
| chr | 4 | NONMMUG072689 | Linc | METAS | UP |
| chr | 4 | NONMMUG072727 | Linc | METAS | UP |
| chr | 4 | NONMMUG093280 | Linc | METAS | UP |
| chr | 4 | NONMMUG031539 | Exonic | METAS | UP |
| chr | 5 | NONMMUG034371 | Exonic | EMT | UP |
|  |  |  |  | MALIG | UP |
| chr | 5 | NONMMUG032788 | Sense no Exonic | MALIG | DOWN |
| chr | 5 | NONMMUG032975 | Exonic | MALIG | DOWN |
| chr | 5 | NONMMUG033312 | Antisense | MALIG | DOWN |
| chr | 5 | NONMMUG033549 | Antisense | MALIG | DOWN |
| chr | 5 | NONMMUG073966 | Linc | METAS | DOWN |
| chr | 5 | NONMMUG032250 | Linc | METAS | DOWN |
| chr | 5 | NONMMUG032747 | Linc | METAS | DOWN |
| chr | 5 | NONMMUG033280 | Sense no Exonic | METAS | DOWN |
| chr | 5 | NONMMUG033910 | Sense no Exonic | METAS | DOWN |
| chr | 5 | NONMMUG034306 | Linc | METAS | DOWN |
| chr | 5 | NONMMUG031822 | Linc | METAS | UP |
| chr | 5 | AI506816 | lncRNA | METAS | UP |
| chr | 5 | NONMMUG075356 | Linc | METAS | UP |
| chr | 5 | NONMMUG032375 | Exonic | METAS | UP |
| chr | 5 | NONMMUG075624 | Linc | METAS | UP |
| chr | 5 | NONMMUG032781 | Exonic | METAS | UP |
| chr | 5 | NONMMUG032929 | Exonic | METAS | UP |
| chr | 5 | NONMMUG033109 | Linc | METAS | UP |
| chr | 5 | NONMMUG033366 | Antisense | METAS | UP |
| chr | 5 | C330018A13Rik | lncRNA | METAS | UP |
| chr | 5 | NONMMUG074972 | Linc | METAS | UP |
| chr | 6 | NONMMUG034674 | Antisense | EMT | DOWN |
| chr | 6 | NONMMUG035585 | Linc | EMT | DOWN |
|  |  |  |  | MALIG | DOWN |
| chr | 6 | NONMMUG077727 | Antisense | EMT | DOWN |
| chr | 6 | NONMMUG035809 | Antisense | EMT | DOWN |
| chr | 6 | NONMMUG036055 | Linc | EMT | DOWN |
| chr | 6 | NONMMUG036111 | Sense no Exonic | EMT | DOWN |
| chr | 6 | NONMMUG036112 | Exonic | EMT | DOWN |
| chr | 6 | NONMMUG036478 | Exonic | EMT | DOWN |
| chr | 6 | NONMMUG036782 | Exonic | EMT | DOWN |
|  |  |  |  | METAS | UP |
| chr | 6 | NONMMUG035357 | Antisense | EMT | UP |
|  |  |  |  | MALIG | UP |
| chr | 6 | NONMMUG035347 | Exonic | MALIG | UP |
| chr | 6 | NONMMUG036638 | Exonic | MALIG | UP |
| chr | 6 | NONMMUG076345 | Linc | METAS | DOWN |
| chr | 6 | NONMMUG034969 | Exonic | METAS | DOWN |
| chr | 6 | NONMMUG034983 | Exonic | METAS | DOWN |
| chr | 6 | NONMMUG035144 | Linc | METAS | DOWN |
| chr | 6 | NONMMUG076838 | Linc | METAS | DOWN |
| chr | 6 | NONMMUG036011 | Linc | METAS | DOWN |
| chr | 6 | NONMMUG036610 | Sense no Exonic | METAS | DOWN |
| chr | 6 | NONMMUG094771 | Sense no Exonic | METAS | DOWN |
| chr | 6 | NONMMUG076323 | Linc | METAS | UP |
| chr | 6 | NONMMUG034529 | Sense no Exonic | METAS | UP |
| chr | 6 | NONMMUG034530 | Sense no Exonic | METAS | UP |
| chr | 6 | NONMMUG034532 | Linc | METAS | UP |
| chr | 6 | NONMMUG034537 | Linc | METAS | UP |
| chr | 6 | NONMMUG076374 | Linc | METAS | UP |
| chr | 6 | NONMMUG034772 | Sense no Exonic | METAS | UP |
| chr | 6 | 2210408F21Rik | lncRNA | METAS | UP |
| chr | 6 | NONMMUG035081 | Linc | METAS | UP |
| chr | 6 | NONMMUG035315 | Linc | METAS | UP |
| chr | 6 | NONMMUG036303 | Antisense | METAS | UP |
| chr | 6 | NONMMUG036584 | Linc | METAS | UP |
| chr | 6 | NONMMUG036585 | Linc | METAS | UP |
| chr | 6 | NONMMUG094443 | Linc | METAS | UP |
| chr | 7 | NONMMUG037484 | Linc | EMT | DOWN |
| chr | 7 | NONMMUG037656 | Linc | EMT | DOWN |
| chr | 7 | NONMMUG038825 | Exonic | MALIG | DOWN |
| chr | 7 | NONMMUG037169 | Linc | METAS | DOWN |
| chr | 7 | NONMMUG078422 | Linc | METAS | DOWN |
| chr | 7 | NONMMUG038280 | Sense no Exonic | METAS | DOWN |
| chr | 7 | NONMMUG039356 | Antisense | METAS | DOWN |
| chr | 7 | NONMMUG039685 | Antisense | METAS | DOWN |
| chr | 7 | NONMMUG078130 | Linc | METAS | UP |
| chr | 7 | NONMMUG037518 | Linc | METAS | UP |
| chr | 7 | NONMMUG037974 | Exonic | METAS | UP |
| chr | 7 | NONMMUG038093 | Linc | METAS | UP |
| chr | 7 | NONMMUG038156 | Linc | METAS | UP |
| chr | 7 | NONMMUG080056 | Linc | METAS | UP |
| chr | 7 | NONMMUG080057 | Linc | METAS | UP |
| chr | 7 | NONMMUG079196 | Linc | METAS | UP |
| chr | 7 | NONMMUG039413 | Sense no Exonic | METAS | UP |
| chr | 8 | NONMMUG040620 | Exonic | EMT | DOWN |
| chr | 8 | NONMMUG081006 | Linc | EMT | DOWN |
| chr | 8 | NONMMUG041885 | Linc | EMT | DOWN |
| chr | 8 | Gm42047 | lncRNA | EMT | UP |
| chr | 8 | NONMMUG040934 | Antisense | MALIG | DOWN |
| chr | 8 | NONMMUG081972 | Linc | METAS | DOWN |
| chr | 8 | NONMMUG040640 | Linc | METAS | DOWN |
| chr | 8 | Gm10033 | Processed transcript | METAS | DOWN |
| chr | 8 | Gm45899 | lncRNA | METAS | DOWN |
| chr | 8 | NONMMUG041516 | Exonic | METAS | DOWN |
| chr | 8 | NONMMUG041687 | Exonic | METAS | DOWN |
| chr | 8 | NONMMUG041761 | Linc | METAS | DOWN |
| chr | 8 | NONMMUG039970 | Linc | METAS | UP |
| chr | 8 | NONMMUG040211 | Linc | METAS | UP |
| chr | 8 | NONMMUG040267 | Exonic | METAS | UP |
| chr | 8 | NONMMUG095762 | Linc | METAS | UP |
| chr | 8 | NONMMUG095519 | Linc | METAS | UP |
| chr | 8 | NONMMUG040711 | Linc | METAS | UP |
| chr | 8 | NONMMUG041144 | Exonic | METAS | UP |
| chr | 8 | NONMMUG081486 | Linc | METAS | UP |
| chr | 8 | NONMMUG095675 | Linc | METAS | UP |
| chr | 9 | NONMMUG043682 | Antisense | EMT | DOWN |
| chr | 9 | NONMMUG043940 | Antisense | EMT | DOWN |
| chr | 9 | 4930461G14Rik | lncRNA | EMT | UP |
| chr | 9 | NONMMUG043691 | Linc | EMT | UP |
|  |  |  |  | MALIG | UP |
| chr | 9 | NONMMUG084653 | Linc | MALIG | DOWN |
| chr | 9 | Gm9531 | Processed transcript | MALIG | UP |
| chr | 9 | NONMMUG042010 | Linc | METAS | DOWN |
| chr | 9 | NONMMUG083078 | Linc | METAS | DOWN |
| chr | 9 | Mir100hg | lncRNA | METAS | DOWN |
| chr | 9 | NONMMUG042550 | Linc | METAS | DOWN |
| chr | 9 | NONMMUG042548 | Linc | METAS | DOWN |
| chr | 9 | NONMMUG042574 | Linc | METAS | DOWN |
| chr | 9 | NONMMUG084600 | Linc | METAS | DOWN |
| chr | 9 | NONMMUG042613 | Linc | METAS | DOWN |
| chr | 9 | NONMMUG042614 | Linc | METAS | DOWN |
| chr | 9 | NONMMUG042674 | Sense no Exonic | METAS | DOWN |
| chr | 9 | NONMMUG042681 | Sense no Exonic | METAS | DOWN |
| chr | 9 | NONMMUG042714 | Linc | METAS | DOWN |
| chr | 9 | NONMMUG043117 | Linc | METAS | DOWN |
| chr | 9 | NONMMUG084882 | Linc | METAS | DOWN |
| chr | 9 | NONMMUG042232 | Exonic | METAS | UP |
| chr | 9 | NONMMUG084497 | Linc | METAS | UP |
| chr | 9 | NONMMUG084499 | Linc | METAS | UP |
| chr | 9 | NONMMUG042534 | Exonic | METAS | UP |
| chr | 9 | NONMMUG096085 | Linc | METAS | UP |
| chr | 9 | NONMMUG043191 | Sense no Exonic | METAS | UP |
| chr | 9 | NONMMUG043328 | Linc | METAS | UP |
| chr | 9 | NONMMUG085249 | Linc | METAS | UP |
| chr | 9 | NONMMUG084029 | Linc | METAS | UP |
| chr | 9 | NONMMUG044238 | Linc | METAS | UP |
| chr | 9 | NONMMUG044343 | Linc | METAS | UP |
| chr | 10 | NONMMUG050805 | Linc | METAS | DOWN |
| chr | 10 | NONMMUG003396 | Linc | METAS | DOWN |
| chr | 10 | NONMMUG051177 | Linc | METAS | DOWN |
| chr | 10 | NONMMUG051178 | Linc | METAS | DOWN |
| chr | 10 | NONMMUG004760 | Linc | METAS | DOWN |
| chr | 10 | NONMMUG003191 | Linc | METAS | UP |
| chr | 10 | NONMMUG003196 | Linc | METAS | UP |
| chr | 10 | NONMMUG003214 | Linc | METAS | UP |
| chr | 10 | NONMMUG003654 | Linc | METAS | UP |
| chr | 10 | NONMMUG003708 | Linc | METAS | UP |
| chr | 10 | NONMMUG003763 | Linc | METAS | UP |
| chr | 10 | NONMMUG051210 | Linc | METAS | UP |
| chr | 10 | NONMMUG004180 | Linc | METAS | UP |
| chr | 10 | NONMMUG004241 | Linc | METAS | UP |
| chr | 10 | NONMMUG051350 | Linc | METAS | UP |
| chr | 10 | NONMMUG051351 | Linc | METAS | UP |
| chr | 10 | NONMMUG004995 | Linc | METAS | UP |
| chr | 11 | NONMMUG005701 | Linc | EMT | UP |
| chr | 11 | NONMMUG005765 | Linc | EMT | UP |
| chr | 11 | NONMMUG006923 | Linc | EMT | UP |
| chr | 11 | NONMMUG007059 | Linc | EMT | UP |
| chr | 11 | NONMMUG007358 | Linc | EMT | UP |
| chr | 11 | NONMMUG007404 | Linc | EMT | UP |
| chr | 11 | NONMMUG007405 | Linc | EMT | UP |
| chr | 11 | NONMMUG007415 | Linc | EMT | UP |
| chr | 11 | NONMMUG007610 | Linc | EMT | UP |
| chr | 11 | NONMMUG006067 | Linc | METAS | DOWN |
| chr | 11 | Olfr1372-ps1 | Processed transcript | METAS | DOWN |
| chr | 11 | NONMMUG007011 | Linc | METAS | DOWN |
| chr | 11 | NONMMUG007141 | Linc | METAS | DOWN |
| chr | 11 | NONMMUG007448 | Linc | METAS | DOWN |
| chr | 11 | NONMMUG005219 | Linc | METAS | UP |
| chr | 11 | Snhg15 | lncRNA | METAS | UP |
| chr | 11 | NONMMUG005345 | Linc | METAS | UP |
| chr | 11 | NONMMUG005721 | Linc | METAS | UP |
| chr | 11 | 4933415A04Rik | lncRNA | METAS | UP |
| chr | 11 | NONMMUG005976 | Linc | METAS | UP |
| chr | 11 | NONMMUG006050 | Linc | METAS | UP |
| chr | 11 | NONMMUG052084 | Linc | METAS | UP |
| chr | 11 | NONMMUG006346 | Linc | METAS | UP |
| chr | 11 | NONMMUG053148 | Linc | METAS | UP |
| chr | 11 | Gm11427 | Processed transcript | METAS | UP |
| chr | 11 | NONMMUG007014 | Linc | METAS | UP |
| chr | 11 | NONMMUG007171 | Linc | METAS | UP |
| chr | 11 | NONMMUG007554 | Linc | METAS | UP |
| chr | 11 | NONMMUG052545 | Linc | METAS | UP |
| chr | 11 | NONMMUG089034 | Linc | METAS | UP |
| chr | 11 | NONMMUG007819 | Linc | METAS | UP |
| chr | 11 | NONMMUG007952 | Linc | METAS | UP |
| chr | 11 | NONMMUG008067 | Linc | METAS | UP |
| chr | 11 | NONMMUG089078 | Linc | METAS | UP |
| chr | 12 | NONMMUG008775 | Linc | MALIG | DOWN |
| chr | 12 | NONMMUG055082 | Linc | METAS | DOWN |
| chr | 12 | NONMMUG055083 | Linc | METAS | DOWN |
| chr | 12 | NONMMUG055084 | Linc | METAS | DOWN |
| chr | 12 | NONMMUG055192 | Linc | METAS | DOWN |
| chr | 12 | NONMMUG055193 | Linc | METAS | DOWN |
| chr | 12 | Gm49327 | Processed transcript | METAS | UP |
| chr | 12 | NONMMUG053817 | Linc | METAS | UP |
| chr | 12 | 1700030C10Rik | Processed transcript | METAS | UP |
| chr | 12 | 3110053B16Rik | Processed transcript | METAS | UP |
| chr | 12 | NONMMUG089378 | Linc | METAS | UP |
| chr | 12 | NONMMUG008678 | Linc | METAS | UP |
| chr | 12 | NONMMUG089188 | Linc | METAS | UP |
| chr | 12 | NONMMUG009325 | Linc | METAS | UP |
| chr | 12 | NONMMUG054375 | Linc | METAS | UP |
| chr | 12 | NONMMUG009847 | Linc | METAS | UP |
| chr | 12 | NONMMUG009943 | Linc | METAS | UP |
| chr | 12 | Gm30948 | lncRNA | METAS | UP |
| chr | 13 | Gpr137b-ps | Processed transcript | EMT | DOWN |
|  |  |  |  | METAS | UP |
| chr | 13 | NONMMUG010281 | Linc | EMT | UP |
| chr | 13 | Gm48682 | lncRNA | EMT | UP |
| chr | 13 | NONMMUG010899 | Linc | EMT | UP |
| chr | 13 | NONMMUG011236 | Linc | EMT | UP |
| chr | 13 | Gm47486 | lncRNA | METAS | DOWN |
| chr | 13 | Gm48754 | lncRNA | METAS | DOWN |
| chr | 13 | Gm48799 | lncRNA | METAS | DOWN |
| chr | 13 | NONMMUG010423 | Linc | METAS | DOWN |
| chr | 13 | NONMMUG056722 | Linc | METAS | DOWN |
| chr | 13 | NONMMUG056723 | Linc | METAS | DOWN |
| chr | 13 | NONMMUG056949 | Linc | METAS | DOWN |
| chr | 13 | NONMMUG010169 | Linc | METAS | UP |
| chr | 13 | 2810429I04Rik | lncRNA | METAS | UP |
| chr | 13 | NONMMUG010211 | Linc | METAS | UP |
| chr | 13 | NONMMUG010555 | Linc | METAS | UP |
| chr | 13 | NONMMUG089618 | Linc | METAS | UP |
| chr | 13 | NONMMUG089844 | Linc | METAS | UP |
| chr | 13 | NONMMUG011033 | Linc | METAS | UP |
| chr | 13 | NONMMUG011252 | Linc | METAS | UP |
| chr | 13 | NONMMUG011344 | Linc | METAS | UP |
| chr | 13 | 3110070M22Rik | lncRNA | METAS | UP |
| chr | 14 | NONMMUG012806 | Linc | EMT | UP |
| chr | 14 | Gm43305 | lncRNA | EMT | UP |
| chr | 14 | NONMMUG013015 | Linc | EMT | UP |
| chr | 14 | Gm20687 | lncRNA | EMT | UP |
| chr | 14 | NONMMUG012182 | Linc | MALIG | DOWN |
| chr | 14 | NONMMUG057571 | Linc | MALIG | DOWN |
| chr | 14 | 9330188P03Rik | lncRNA | MALIG | DOWN |
| chr | 14 | NONMMUG012176 | Linc | METAS | DOWN |
| chr | 14 | NONMMUG012433 | Linc | METAS | DOWN |
| chr | 14 | NONMMUG012443 | Linc | METAS | DOWN |
| chr | 14 | NONMMUG012555 | Linc | METAS | DOWN |
| chr | 14 | NONMMUG012931 | Linc | METAS | DOWN |
| chr | 14 | NONMMUG013142 | Linc | METAS | DOWN |
| chr | 14 | NONMMUG090316 | Linc | METAS | DOWN |
| chr | 14 | NONMMUG013173 | Linc | METAS | DOWN |
| chr | 14 | NONMMUG013305 | Linc | METAS | DOWN |
| chr | 14 | NONMMUG013390 | Linc | METAS | DOWN |
| chr | 14 | Gm10110 | Processed transcript | METAS | DOWN |
| chr | 14 | NONMMUG013880 | Linc | METAS | DOWN |
| chr | 14 | Gm31517 | lncRNA | METAS | UP |
| chr | 14 | NONMMUG012801 | Linc | METAS | UP |
| chr | 14 | NONMMUG013259 | Linc | METAS | UP |
| chr | 14 | NONMMUG013286 | Linc | METAS | UP |
| chr | 14 | NONMMUG013551 | Linc | METAS | UP |
| chr | 14 | NONMMUG059207 | Linc | METAS | UP |
| chr | 14 | NONMMUG013808 | Linc | METAS | UP |
| chr | 15 | NONMMUG060759 | Linc | EMT | DOWN |
| chr | 15 | NONMMUG060945 | Linc | EMT | DOWN |
| chr | 15 | NONMMUG060946 | Linc | EMT | DOWN |
| chr | 15 | NONMMUG015514 | Linc | EMT | DOWN |
|  |  |  |  | METAS | UP |
| chr | 15 | Gm48957 | lncRNA | EMT | UP |
| chr | 15 | NONMMUG014171 | Linc | EMT | UP |
| chr | 15 | NONMMUG014649 | Linc | EMT | UP |
| chr | 15 | NONMMUG014882 | Linc | EMT | UP |
| chr | 15 | NONMMUG015352 | Linc | EMT | UP |
| chr | 15 | NONMMUG015380 | Linc | EMT | UP |
| chr | 15 | NONMMUG015565 | Linc | EMT | UP |
| chr | 15 | NONMMUG014060 | Linc | METAS | DOWN |
| chr | 15 | NONMMUG014061 | Linc | METAS | DOWN |
| chr | 15 | NONMMUG014496 | Linc | METAS | DOWN |
| chr | 15 | NONMMUG014497 | Linc | METAS | DOWN |
| chr | 15 | NONMMUG014501 | Linc | METAS | DOWN |
| chr | 15 | NONMMUG014605 | Linc | METAS | DOWN |
| chr | 15 | NONMMUG014607 | Linc | METAS | DOWN |
| chr | 15 | NONMMUG014613 | Linc | METAS | DOWN |
| chr | 15 | NONMMUG059968 | Linc | METAS | DOWN |
| chr | 15 | NONMMUG014784 | Linc | METAS | DOWN |
| chr | 15 | NONMMUG014888 | Linc | METAS | DOWN |
| chr | 15 | NONMMUG014959 | Linc | METAS | DOWN |
| chr | 15 | NONMMUG014968 | Linc | METAS | DOWN |
| chr | 15 | NONMMUG059440 | Linc | METAS | UP |
| chr | 15 | NONMMUG014167 | Linc | METAS | UP |
| chr | 15 | NONMMUG059735 | Linc | METAS | UP |
| chr | 15 | NONMMUG059955 | Linc | METAS | UP |
| chr | 15 | Gm49497 | lncRNA | METAS | UP |
| chr | 15 | NONMMUG014636 | Linc | METAS | UP |
| chr | 15 | NONMMUG014744 | Linc | METAS | UP |
| chr | 15 | NONMMUG060096 | Linc | METAS | UP |
| chr | 15 | NONMMUG015364 | Linc | METAS | UP |
| chr | 15 | NONMMUG061049 | Linc | METAS | UP |
| chr | 15 | NONMMUG015409 | Linc | METAS | UP |
| chr | 16 | Mx1 | Processed transcript | EMT | DOWN |
|  |  |  |  | MALIG | DOWN |
| chr | 16 | NONMMUG016086 | Linc | EMT | UP |
| chr | 16 | NONMMUG016679 | Linc | EMT | UP |
| chr | 16 | NONMMUG017258 | Linc | EMT | UP |
| chr | 16 | NONMMUG016694 | Linc | MALIG | DOWN |
| chr | 16 | NONMMUG016698 | Linc | MALIG | DOWN |
| chr | 16 | NONMMUG016700 | Linc | MALIG | DOWN |
| chr | 16 | NONMMUG016702 | Linc | MALIG | DOWN |
| chr | 16 | NONMMUG017293 | Linc | MALIG | DOWN |
| chr | 16 | NONMMUG016182 | Linc | METAS | DOWN |
| chr | 16 | NONMMUG016322 | Linc | METAS | DOWN |
| chr | 16 | NONMMUG016861 | Linc | METAS | DOWN |
| chr | 16 | NONMMUG017042 | Linc | METAS | DOWN |
| chr | 16 | NONMMUG017150 | Linc | METAS | DOWN |
| chr | 16 | NONMMUG017180 | Linc | METAS | DOWN |
| chr | 16 | NONMMUG017182 | Linc | METAS | DOWN |
| chr | 16 | NONMMUG016352 | Linc | METAS | UP |
| chr | 16 | NONMMUG016391 | Linc | METAS | UP |
| chr | 16 | Gm49701 | lncRNA | METAS | UP |
| chr | 16 | NONMMUG016826 | Linc | METAS | UP |
| chr | 16 | NONMMUG017009 | Linc | METAS | UP |
| chr | 17 | NONMMUG091337 | Linc | EMT | DOWN |
| chr | 17 | NONMMUG017999 | Linc | EMT | UP |
| chr | 17 | NONMMUG018123 | Linc | EMT | UP |
| chr | 17 | NONMMUG018213 | Linc | EMT | UP |
| chr | 17 | NONMMUG019046 | Linc | EMT | UP |
| chr | 17 | NONMMUG019129 | Linc | EMT | UP |
| chr | 17 | NONMMUG017903 | Linc | MALIG | DOWN |
| chr | 17 | NONMMUG017389 | Linc | METAS | DOWN |
| chr | 17 | NONMMUG017413 | Linc | METAS | DOWN |
| chr | 17 | NONMMUG063023 | Linc | METAS | DOWN |
| chr | 17 | NONMMUG017678 | Linc | METAS | DOWN |
| chr | 17 | NONMMUG017995 | Linc | METAS | DOWN |
| chr | 17 | NONMMUG018156 | Linc | METAS | DOWN |
| chr | 17 | NONMMUG018812 | Linc | METAS | DOWN |
| chr | 17 | NONMMUG018814 | Linc | METAS | DOWN |
| chr | 17 | NONMMUG091316 | Linc | METAS | DOWN |
| chr | 17 | NONMMUG018344 | Linc | METAS | UP |
| chr | 17 | NONMMUG064070 | Linc | METAS | UP |
| chr | 17 | NONMMUG018843 | Linc | METAS | UP |
| chr | 17 | Trmt61b | lncRNA | METAS | UP |
| chr | 18 | NONMMUG019417 | Linc | EMT | DOWN |
|  |  |  |  | MALIG | DOWN |
| chr | 18 | NONMMUG020007 | Linc | EMT | UP |
| chr | 18 | NONMMUG020083 | Linc | EMT | UP |
| chr | 18 | NONMMUG020347 | Linc | EMT | UP |
| chr | 18 | NONMMUG019357 | Linc | MALIG | DOWN |
| chr | 18 | NONMMUG019647 | Linc | MALIG | DOWN |
| chr | 18 | NONMMUG019483 | Linc | METAS | DOWN |
| chr | 18 | NONMMUG019487 | Linc | METAS | DOWN |
| chr | 18 | Zscan30 | Processed transcript | METAS | DOWN |
| chr | 18 | NONMMUG019566 | Linc | METAS | DOWN |
| chr | 18 | NONMMUG019722 | Linc | METAS | DOWN |
| chr | 18 | NONMMUG064790 | Linc | METAS | DOWN |
| chr | 18 | NONMMUG019543 | Linc | METAS | UP |
| chr | 18 | NONMMUG064786 | Linc | METAS | UP |
| chr | 18 | Gm41760 | lncRNA | METAS | UP |
| chr | 18 | AC132307.1 | lncRNA | METAS | UP |
| chr | 19 | NONMMUG021256 | Linc | EMT | UP |
| chr | 19 | NONMMUG021327 | Linc | EMT | UP |
| chr | 19 | NONMMUG021331 | Linc | EMT | UP |
| chr | 19 | NONMMUG021544 | Linc | EMT | UP |
| chr | 19 | NONMMUG021602 | Linc | EMT | UP |
| chr | 19 | NONMMUG021688 | Linc | EMT | UP |
| chr | 19 | NONMMUG020553 | Linc | METAS | DOWN |
| chr | 19 | NONMMUG020586 | Linc | METAS | DOWN |
| chr | 19 | NONMMUG020673 | Linc | METAS | DOWN |
| chr | 19 | NONMMUG065760 | Linc | METAS | DOWN |
| chr | 19 | NONMMUG020855 | Linc | METAS | DOWN |
| chr | 19 | Gm50216 | lncRNA | METAS | DOWN |
| chr | 19 | NONMMUG066351 | Linc | METAS | DOWN |
| chr | 19 | NONMMUG065931 | Linc | METAS | DOWN |
| chr | 19 | NONMMUG092026 | Linc | METAS | DOWN |
| chr | 19 | NONMMUG091924 | Linc | METAS | UP |
| chr | 19 | NONMMUG020717 | Linc | METAS | UP |
| chr | 19 | Gm14964 | lncRNA | METAS | UP |
| chr | 19 | NONMMUG021044 | Linc | METAS | UP |
| chr | 19 | NONMMUG021229 | Linc | METAS | UP |
| chr | 19 | NONMMUG021352 | Linc | METAS | UP |
| chr | 19 | NONMMUG091984 | Linc | METAS | UP |
| chr | X | NONMMUG044486 | Linc | EMT | DOWN |
| chr | X | NONMMUG044909 | Linc | EMT | DOWN |
| chr | X | Xist | lncRNA | MALIG | DOWN |
| chr | X | NONMMUG045569 | Linc | MALIG | DOWN |
| chr | X | NONMMUG044425 | Linc | METAS | DOWN |
| chr | X | NONMMUG044889 | Linc | METAS | DOWN |
| chr | X | NONMMUG096512 | Linc | METAS | DOWN |
| chr | X | NONMMUG045574 | Linc | METAS | UP |
| chr | X | NONMMUG045946 | Linc | METAS | UP |
| chr | X | NONMMUG045988 | Linc | METAS | UP |
| chr | X | NONMMUG046218 | Linc | METAS | UP |
| chr | X | NONMMUG046220 | Linc | METAS | UP |
| chr | Y | NONMMUG096601 | Linc | METAS | DOWN |
| chr | Y | Gm47283 | lncRNA | METAS | DOWN |
| chr | Y | NONMMUG046461 | Linc | METAS | UP |

**Table 4. Enriched pathways**

**Figure S1**

**
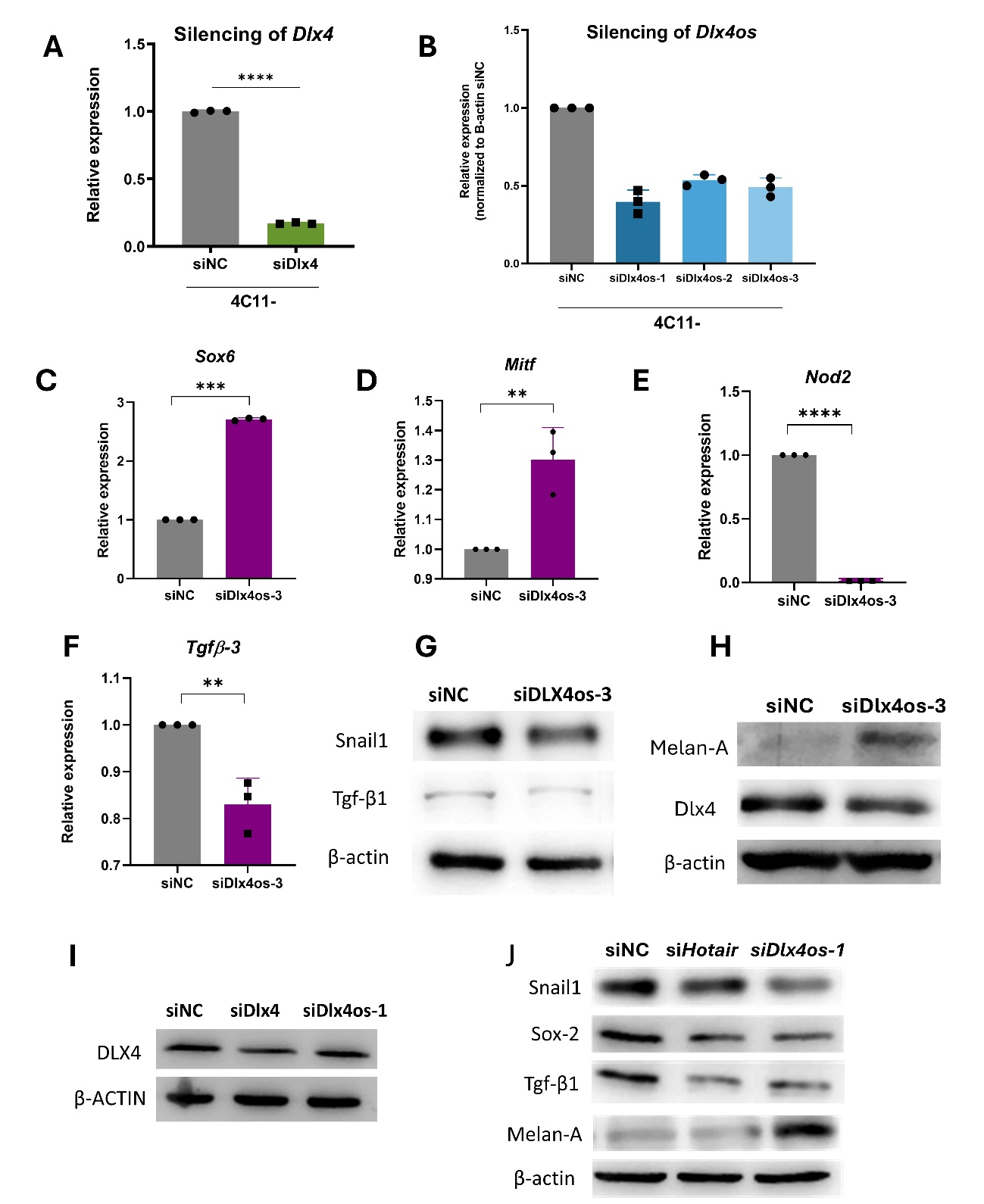
**

**Figure S1. *Dlx4os* knockdown promotes a less malignant phenotype in melanoma cells. A.** The expression of *Dlx4os* was evaluated in 4C11- cells transfected or not with siRNA, using RT-qPCR, relative to negative control cells (siNC), with *Rpl19* used as a reference gene. **B.** The expression of *Dlx4os* was evaluated in 4C11- cells transfected with three different siRNA sequences (siDlx4os-1, siDlx4os-2, siDlx4os-3) or not, using RT-qPCR relative to negative control cells (siNC), with *Rpl19* as a reference gene. **C–F.** *Dlx4os* silencing increased the expression of *Sox6* and *Mitf*, and decreased the expression of *Nod2* and *Tgfβ3*, as measured by qPCR and normalized to *β-actin* and the negative control cells (siNC). **G–I.** Representative Western blot images showing the expression of Melan-A, DLX4, SNAIL1, and TGF-β1 upon *Dlx4os* silencing in 4C11- cells transfected with different siRNA sequences. *β-actin* was used as a loading control. **J.** Full gel image corresponding to the image in Figure 3D. The data are presented as mean ± SD values (n ≥ 3). *p-value < 0.05; **p-value < 0.01; ***p-value < 0.001

**Figure S2**

**
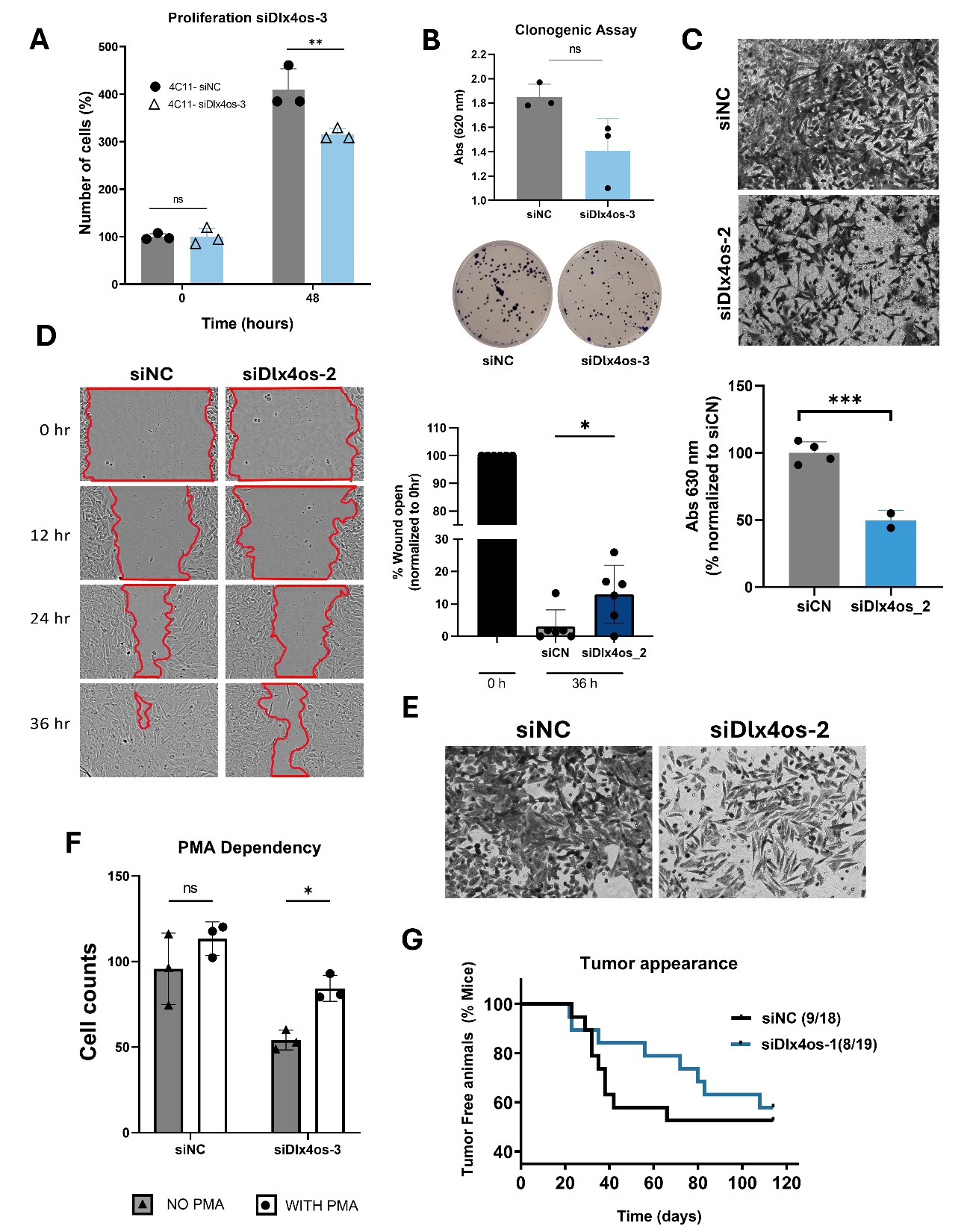
**

**Figure S2. *Dlx4os* knockdown promotes a less malignant phenotype in melanoma cells**. **A.** Cell proliferation was assessed by counting viable cells after 48 hours of silencing, performed manually using a Neubauer chamber. Percentage relative to the control (siNC). **B.** Quantification of colonies estimated by the absorbance of the solution after lysing cells stained with Toluidine blue using ImageJ 1.52q software (down), and photographic representation of colonies stained with Toluidine Blue (up). **C.** Quantification of Toluidine Blue dye absorbance shown in the graph (down) and image of individual migration after 4 hours, in laminin-coated inserts, in serum-free medium (10 μg/ml; images at 20X magnification) (up) **D.** Representative image of scratch wounds (10X) at 0, 12, and 24 hours analyzed using ImageJ 1.52q software (left) and quantification of the open area (mm²) 36 hours after wounding (right). **E.** Cell invasion relative to the control (siNC) after 64 hours, in laminin-coated inserts (10 μg/ml; images at 20X magnification) in RPMI medium supplemented with 5% serum. **F.** Cell culture performed in the absence (NO PMA – gray) or presence (WITH PMA – white) of PMA in 4C11- **G.** Graph of *in vivo* tumor appearance for *Dlx4os* silencing versus control. The data are presented as mean ± SD values (n ≥ 3). *P < 0.05; **P < 0.01; ***P < 0.001

**Figure S3.**

**
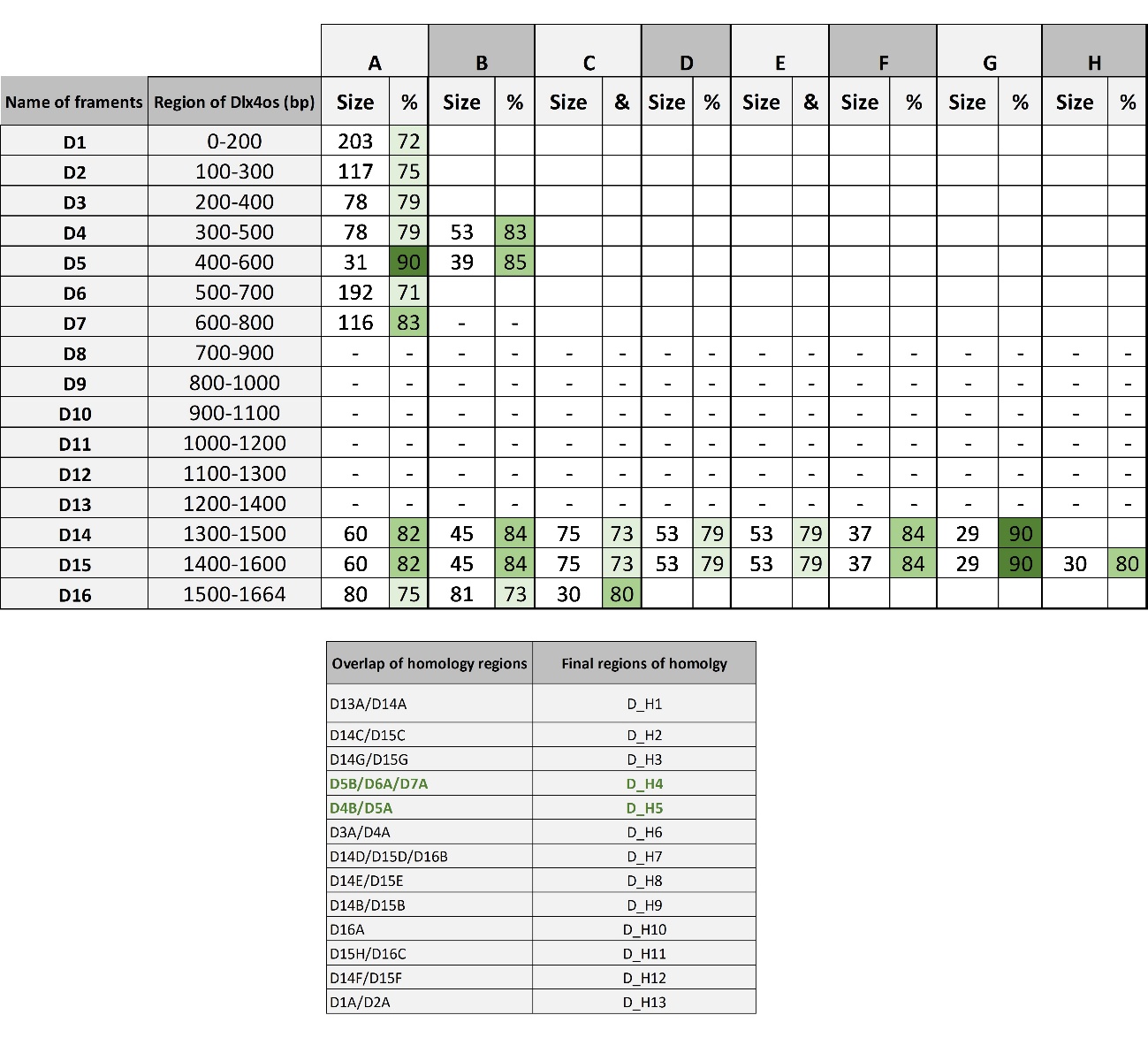
**

**Figure S3. Search for homology on the alignment of *Dlx4os* murine and human sequences on BLAST.** Using 16 fragments of Dlx4os, their alignment with the human genome was observed, based on size (bp) and percentage of alignment (%); the fragments were aligned to observe the intersections and were identified as 13 homologous regions (final homology regions). The regions D_H4 and D_H5 (green) were the regions that were aligned on both LiftGenome and HSALNT0242265 regions.
