# Supplementary material for: LncRNA Dlx4os drives malignant transformation and phenotype switching in melanoma
Source: Epigenetics. 2026 Mar 19;21(1):2641924. doi: 10.1080/15592294.2026.2641924 (PMC13003849; doi:10.1080/15592294.2026.2641924)
Supplement: Table S2.docx [file KEPI_A_2641924_SM4552.docx]

| **Primary** | | |  | **Secundary** | |
| --- | --- | --- | --- | --- | --- |
| **Antibody** | **Catalog** | **Molecular weigh** |  | **Antibody** | **Catalog** |
| vinculin | Cell Signaling #4650 | 128 kDa |  | anti-rabbit | KPL #04-15-06 |
| lamin A/C | Santa Cruz #6215 | 68-72 kDa |  | anti-goat | KPL #14-13-06 |
| Snail | Cell Signalling #3879 | 29kDa |  | anti-rabbit | KPL #04-15-06 |
| Sox2 | Abgent AM2048A | 38kDa |  | anti-mouse | Biorad #1706516 |
| TgB1 | Santa Cruz #130348 | 25/14 kDa |  | anti-rabbit | KPL #04-15-06 |
| mlana | Santa Cruz #20032 | 23kDa |  | anti-mouse | Biorad #1706516 |
| B-actina | Abgene AM1829b | 42 kDa |  | anti-mouse | Biorad #1706516 |
