# Supplementary material for: LncRNA Dlx4os drives malignant transformation and phenotype switching in melanoma
Source: Epigenetics. 2026 Mar 19;21(1):2641924. doi: 10.1080/15592294.2026.2641924 (PMC13003849; doi:10.1080/15592294.2026.2641924)
Supplement: Table S1.docx [file KEPI_A_2641924_SM4551.docx]

| **Primer** | **Specie** | **Foward** | **Reverse** |
| --- | --- | --- | --- |
| Beta-actina | mouse | ACCGTGAAAAGATGACCAG | GTACGCCAGACGCATACAG |
| Dlx4OS | mouse | CTAATCCTGCCTCCACCCTG | CTCCATGAGGGTCTGTTGGT |
| Mitf | mouse | CCTATGGCTATGCTCACTCTT | GTTCATACCTGGGCACTCAC |
| Nod2 | mouse | CCTAGCACTGATGCTGGAGAAG | CGGTAGGTGATGCCATTGTTGG |
| Sox10 | mouse | TCAGAGTTAGCATGGCACGG | AAGGGTGCAAGGCAAAGGTA |
| Sox6 | mouse | GCATAAGTGACCGTTTTGGCAGG | GGCATCTTTGCTCCAGGTGACA |
| Tgfb3 | mouse | AAGCAGCGCTACATAGGTGGCA | GGCTGAAAGGTGTGACATGGAC |
| U6 | mouse | AGGGCTACCCAGTGTTCTGA | TGTGGAACACTACATGAATTTGC |
| Rpl19 | mouse | GAAATCGCCAATGCCAACTC | CTTCCCTATGCCCATATG CC |
| mlana | mouse | GACGAAGTGGATACAGAACCTTG | CTCTTGAGAAGACAGTCGGCTG |
| GAPDH | human | ACCTGACCTGCCGTCTAGAA | GTCAAAGGTGGAGGAGTGGG |
| HSALNT | human | AGGGCCATGAAACTTTGAGGAGC | ACAAGCCCTTAACCAGCCCAA |
