# Supplementary material for: LncRNA Dlx4os drives malignant transformation and phenotype switching in melanoma
Source: Epigenetics. 2026 Mar 19;21(1):2641924. doi: 10.1080/15592294.2026.2641924 (PMC13003849; doi:10.1080/15592294.2026.2641924)
Supplement: Supplementary file 6 [file KEPI_A_2641924_SM4557.docx]

Term	Adjusted P-value	Genes
regulation of angiogenesis (GO:0045765)	0.0072	ECM1;SP100;SPARC;SEMA6A;PTGIS;HSPB1;TSPAN12;THBS2;HIF1A;PGF;HIPK2;ERBB2;EMILIN1;PLCG1
regulation of collagen biosynthetic process (GO:0032965)	0.0072	BMP4;HDAC2;TGFB3;EMILIN1;VIM
cellular response to growth factor stimulus (GO:0071363)	0.0072	BMP4;STAT5B;SEMA6A;SMAD3;MAGI2;ERBB2;HSPB1;RAPGEF2;PLCG1;LRP2;SOX6;ACVR1B
positive regulation of intracellular signal transduction (GO:1902533)	0.0133	ECM1;ADGRV1;NOD1;MST1R;NMI;LRP2;NOD2;FKBP1A;WNT11;ADRB3;NRG4;TRAF5;ERBB2;GCNT2;RAPGEF2;F2RL1;CCR7;LGALS9;IKBKG;FGFR3;TMEM106A;MYD88;BCL2L1
positive regulation of interleukin-1 beta production (GO:0032731)	0.0133	P2RX7;HSPB1;F2RL1;LGALS9;NOD2;TMEM106A;MYD88
mature conventional dendritic cell differentiation (GO:0097029)	0.0143	F2RL1;CCR7;LGALS9
regulation of interleukin-1 beta production (GO:0032651)	0.0154	P2RX7;HSPB1;F2RL1;CCR7;LGALS9;NOD2;TMEM106A;MYD88
positive regulation of interleukin-1 production (GO:0032732)	0.0154	P2RX7;HSPB1;F2RL1;LGALS9;NOD2;TMEM106A;MYD88
regulation of I-kappaB kinase/NF-kappaB signaling (GO:0043122)	0.0154	ECM1;RIOK3;HSPB1;NOD1;NOD2;FKBP1A;TRAF5;F2RL1;CCR7;LGALS9;IKBKG;TMEM106A;MYD88
positive regulation of antigen processing and presentation (GO:0002579)	0.0154	CCR7;NOD1;NOD2
positive regulation of dendritic cell antigen processing and presentation (GO:0002606)	0.01548	CCR7;NOD1;NOD2
positive regulation of collagen biosynthetic process (GO:0032967)	0.01781	BMP4;HDAC2;TGFB3;VIM
positive regulation of I-kappaB kinase/NF-kappaB signaling (GO:0043123)	0.01781	FKBP1A;ECM1;TRAF5;F2RL1;CCR7;NOD1;LGALS9;IKBKG;NOD2;MYD88;TMEM106A
extracellular matrix organization (GO:0030198)	0.01781	SPARC;COL24A1;LAMC3;ITGA2;LOXL4;P3H4;ADAMTSL1;MMP11;MMP24;SMOC1;COL6A1;COL9A3;ITGA6;TIMP1;JAM3
positive regulation of collagen metabolic process (GO:0010714)	0.01898	BMP4;HDAC2;TGFB3;VIM
positive regulation of macromolecule metabolic process (GO:0010604)	0.02429	IL33;SMAD3;SLC2A10;MAGI2;MITF;HIF1A;CLU;BMP4;ACTA2;P2RX7;WNT11;DNAJA4;EMILIN1;LGALS9;PPARGC1A;WNT3;MYD88
extracellular structure organization (GO:0043062)	0.02640	ADAMTSL1;MMP11;SPARC;MMP24;COL24A1;LAMC3;SMOC1;ITGA2;COL6A1;COL9A3;ITGA6;JAM3
external encapsulating structure organization (GO:0045229)	0.02640	ADAMTSL1;MMP11;SPARC;MMP24;COL24A1;LAMC3;SMOC1;ITGA2;COL6A1;COL9A3;ITGA6;JAM3
regulation of dendritic cell antigen processing and presentation (GO:0002604)	0.03440	CCR7;NOD1;NOD2
cellular response to muramyl dipeptide (GO:0071225)	0.03440	NOD1;NOD2;VIM
positive regulation of gene expression (GO:0010628)	0.03440	IL33;SMAD3;SLC2A10;MITF;HIF1A;CLU;BMP4;RPS4X;ACTA2;P2RX7;WNT11;DNAJA4;ERBB2;EMILIN1;LGALS9;VIM;PPARGC1A;WNT3;MYD88
negative regulation of transmembrane receptor protein serine/threonine kinase signaling pathway (GO:0090101)	0.03545	FKBP1A;SMAD3;SLC2A10;MAGI2;RBPMS2;EMILIN1;LRP2;HIPK2
myelin maintenance (GO:0043217)	0.04235	PRX;ABCD1;CLU
positive regulation of endothelial cell migration (GO:0010595)	0.04271	BMP4;SPARC;ATOH8;HSPB1;PLCG1;HIF1A;GPLD1
regulation of ERK1 and ERK2 cascade (GO:0070372)	0.04389	SEMA6A;ATP6AP1;ERBB2;GCNT2;RAPGEF2;RANBP9;F2RL1;CCR7;EMILIN1;LGALS9;NOD2;FGFR3
